# Supplementary material for: High efficiency and long-term intracellular activity of an enzymatic nanofactory based on metal-organic frameworks
Source: Nat Commun. 2017 Dec 12;8:2075. doi: 10.1038/s41467-017-02103-0 (PMC5727123; doi:10.1038/s41467-017-02103-0)
Supplement: Supplementary file 2 — Supplementary Information [file 41467_2017_2103_MOESM2_ESM.pdf]

Supplementary Methods

Chemicals, instruments and cell lines

Aluminum chloride hexahydrate, N-bromosuccinimide (NBS), benzoyl peroxide (BPO), ethylenediamine, triethylamine, trifluoroborane etherate, N-hydroxysuccinimide, N-(3-Dimethylaminopropyl)-N'-ethylcarbodiimide (EDC), N,N-dimethylformamide (DMF), tetrahydrofuran (THF), potassium carbonate, hydrochloric acid and sodium hydroxide were purchased from VWR. Carbon tetrachloride, superoxide dismutase (SOD), catalase (CAT), SOD assay, horseradish peroxide (HRP) and Amplex Red were purchased from Sigma Aldrich. Dulbecco's minimum essential medium (DMEM), 2-[4-(2-hydroxyethyl)piperazin-1-yl]ethanesulfonic acid (HEPES), Leibovitz's L-15 medium without cysteine (non-reducing L-15, nrL-15), 10% fetal bovine serum (FBS) and 1× penicillin/streptomycin (P/S), Hoechst 33342, Lyso Tracker red, SYTOX Blue and SYTOX Green were purchased from Thermo Fisher Scientific. TATB and m-BTB were prepared based on the previous report.[1,2] dfTAT used in this paper was resistant to peptide hydrolase and was prepared according to previous method.[3] pH 5.0 buffer is 50 mM sodium citrate buffer. pH 7.4 buffer is 50 mM HEPES buffer.

Synthetic manipulations that required an inert atmosphere (where noted) were carried out under nitrogen using standard Schlenk techniques. Powder X-ray diffraction (PXRD) was carried out on a Bruker D8-Focus Bragg-Brentano X-ray powder Diffractometer equipped with a Cu sealed tube (λ = 1.54178) at 40 kV and 40 mA. N_2 sorption isotherms at 77 K were measured by using a Micrometritics ASAP 2420 system with high-purity grade (99.999%) of gases. UV-Vis spectra were recorded on Shimadzu UV-2450 spectrophotometer. Fluorescence spectra were recorded on a Hitachi F-4600 spectrometer (Hitachi Co. Ltd., Japan) with Xe lamp as the excitation source at room temperature. ICP analysis was conducted on PerkinElmer NexION 300D instrument. Dynamic light scattering and Zeta potential were measured at 25 °C on a Zetasizer Nano ZS ZEN3600 analyser (Malvern Instrument Ltd, UK). Confocal laser scanning microscopy (CLSM) imaging was performed on an inverted epifluorescence microscope (Model IX81, Olympus) was equipped with a heating stage maintained at 37 °C. Images were collected using a Rolera-MGI Plus back-illuminated electron-multiplying charge coupled device (EMCCD) camera (Qimaging). Images were acquired using bright-field imaging and three standard fluorescence filter sets: DAPI (excitation (Ex) = 350 ± 10 nm/emission (Em) = 440 ± 20 nm), RFP (Ex = 560 ± 20 nm/Em = 630 ± 35 nm) and FITC (Ex = 488 ± 10 nm/Em = 520 ± 20 nm). The images were processed with the SlideBook 4.2 software (Olympus) and ImageJ2.[4,5] Inductively coupled plasma mass spectrometry (ICP-MS) was carried out on an Agilent 7700x series ICP-MS instrument. TEM images were taken on a transmission electron microscopy (JEOL JEM-2100F, Japan) operated at an acceleration voltage of 200 keV by dropping solution onto a carbon-coated copper grid.

HeLa (ATCC CCL-2), NIH3T3 (ATCC CRL-1658) and HDF (ATCC PCS-201-012) were grown in DMEM supplemented with FBS and P/S and kept at 37 °C in a humidified atmosphere containing 5% CO_2. Cell experiments are performed in quintuplicates for each condition, and are repeated with three different batches of cells. Statistic tests are studied by multiple t tests method. HeLa cells are common vitro model for oxidative stress studies.[6-8]

Synthesis of BTB-Green

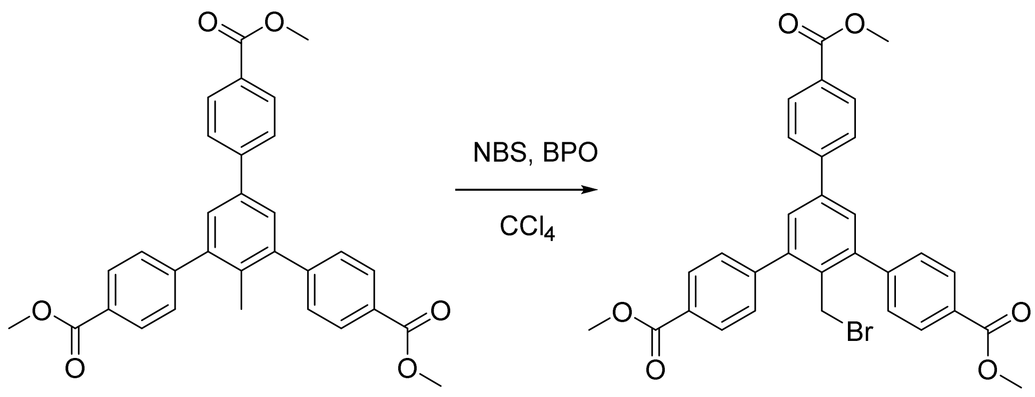

m-BTB **1**

3.68 g m-BTB, 1.35 g NBS and 50 mg BPO was dispersed in 45 mL CCl4 and refluxed at 80°C for 12 h. The precipitate was filtered and the filtrate was dried under vacuum. Compound **1** was obtained as light yellow solid. Yield: 100%.

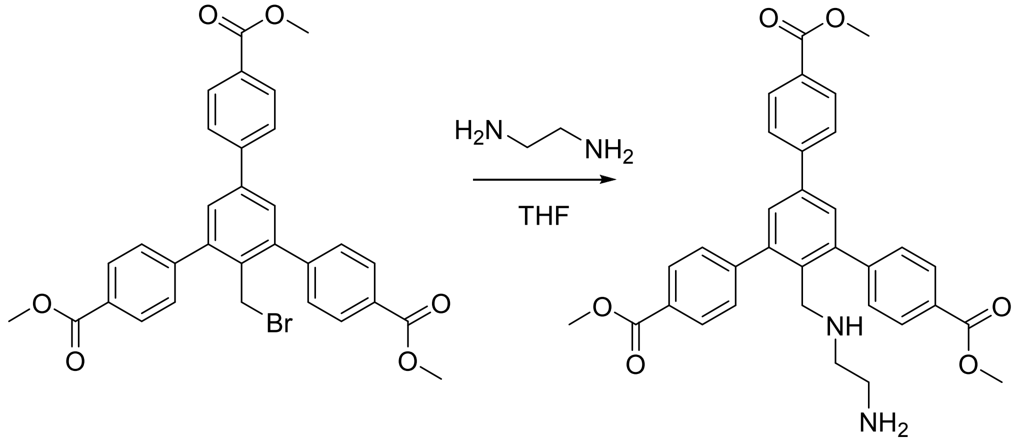

1 **2**

1.6 g compound **1** was dissolved in 60 mL THF. 1 mL ethylenediamine was added and the mixture was stirred under room temperature for 6 h. Saturated $NaHCO_3$ solution was added and the aqueous phase was extracted with CH_2Cl_2 for 3 times. The combined organic phase was dried over $MgSO_4$ and the solvent was removed under vacuum. Compound **2** was obtained as light yellow solid.

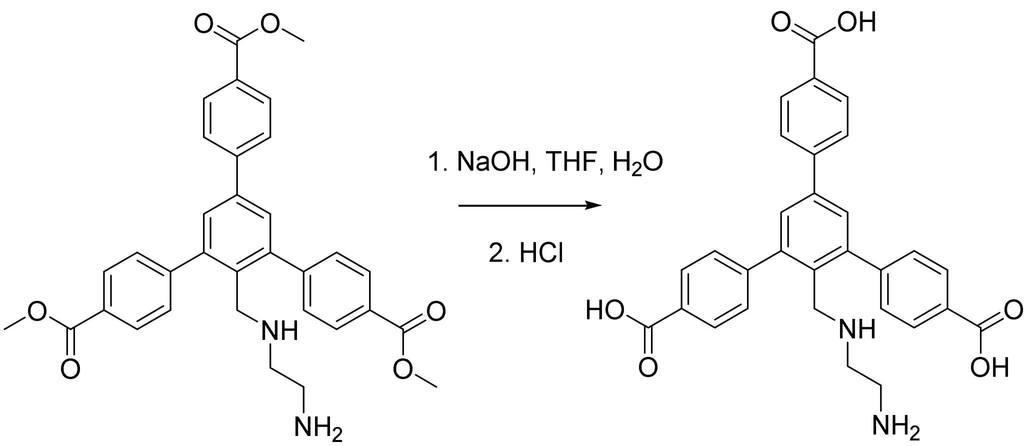

2 **3**

1.8 g compound **2** was dissolved in THF and 20 mL 2 M NaOH solution was added. The mixture was refluxed for 6 h after which THF was removed under vacuum. 6 M HCl was added until compound **3** precipitated as white solid, which was filtered and dried under vacuum.

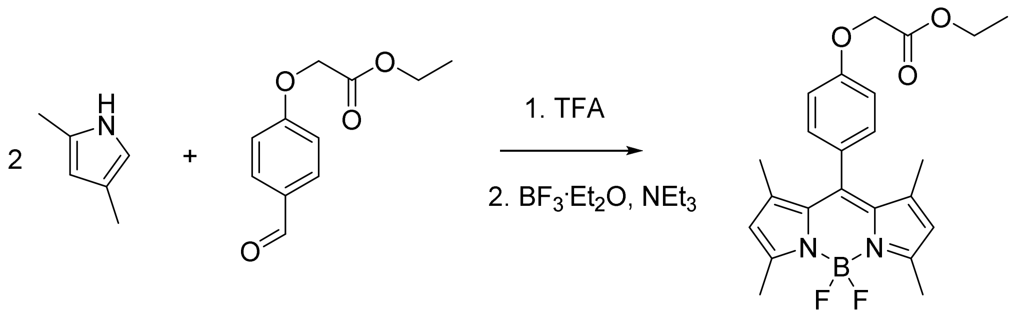

1' **2'**

0.95 g 2, 4-dimethylpyrrole and 1.04 g compound 1’ was dissolved in 150 mL THF. 3 drops of TFA was added and the mixture was stirred overnight at room temperature under darkness, after which 2 g DDQ dissolved in 50 mL THF was added and stirred for 30 minutes. 25 mL triethylamine was added to the mixture, and 31 mL BF3·Et2O was added over 30 minutes. The mixture was stirred for 6 h and compound 2’ was obtained by column chromatography (CH_2Cl_2: hexanes = 1: 2) as an orange solid. Yield: 50%.

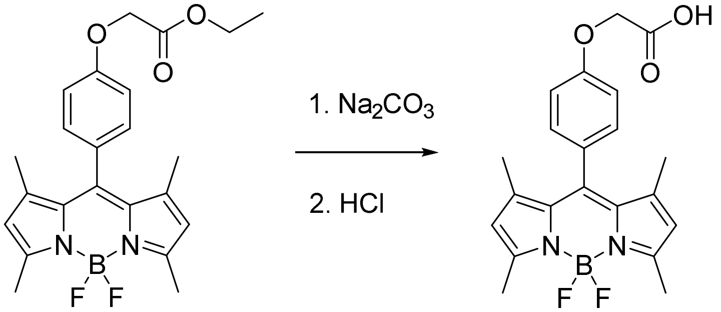

2' **3'**

0.4 g compound 2’ and 0.8 g K_2CO_3 was dissolved in a 1:1 mixture of THF and water. The solution was heated at 50 °C for 24 h, after which the solvent was removed under vacuum. Water was added to the solid and abstracted by CH_2Cl_2 for 3 times in order to remove the unreacted compound 2’. The aqueous phase was added 1 M HCl until pH reached 7. Compound 3’ was obtained by filtration and vacuum drying as an orange solid. Yield: 99%.

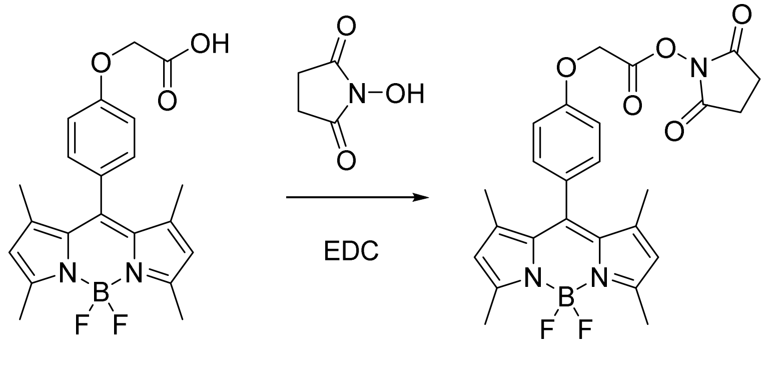

3' **4'**

350 mg compound 3’, 200 mg N-hydroxysuccinimide and 340 mg EDC was suspended in 20 mL CH_2Cl_2 under N_2 atmosphere. The mixture was stirred at room temperature for 12 h and diluted by 200 mL 1:1 CH_2Cl_2/H_2O. The organic phase was dried and compound 4’ was obtained as an orange solid. Yield: 100%.

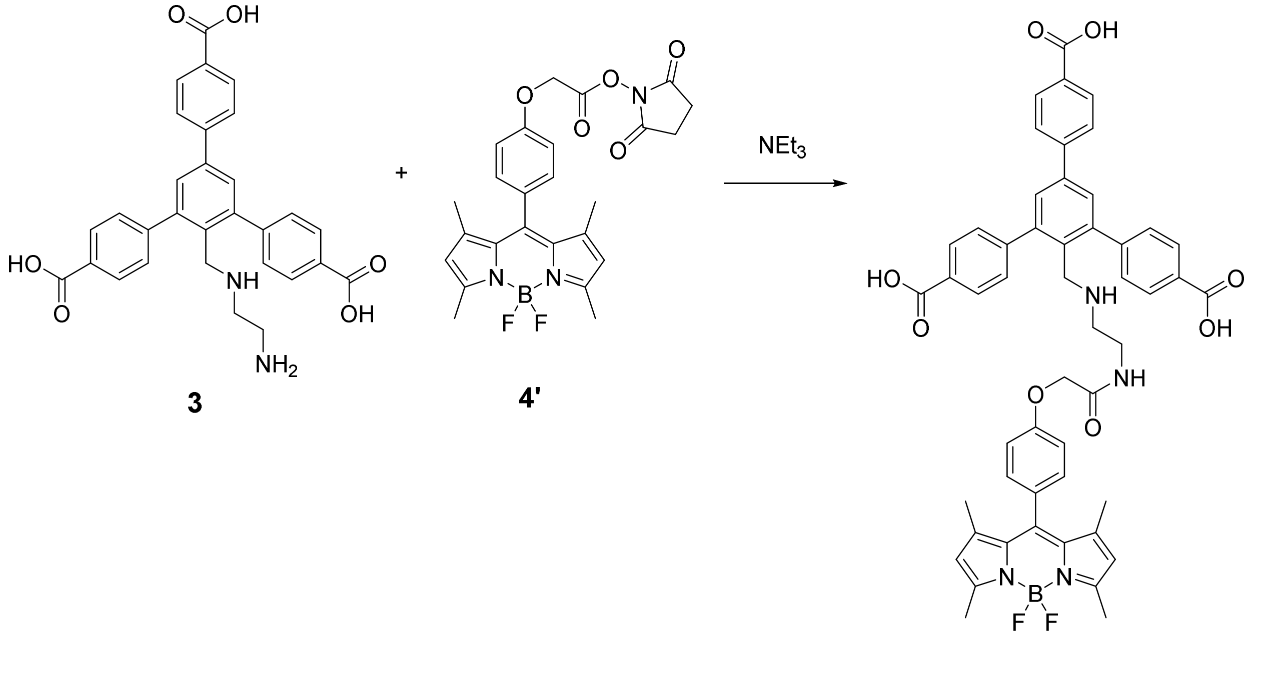

3 **4'**

BTB-Green

510 mg compound 3 and 495 mg compound 4’ was dissolved in 20 mL DMF and 1mL triethylamine was added. The mixture was stirred at room temperature for 12 h and diluted with 100 mL water. 1 M HCl was added until pH reached 4. BTB-Green was obtained by filtration and vacuum drying as an orange solid. Yield: 100%. ESI+: 891.3371.

Synthesis of NPCN-333

10 mL DMF solution of $AlCl_3 \cdot 6H_2O$ (1.5 mg/mL), 5 mL DMF solution of TATB (1 mg/mL), 15 mL DMF and 50 µL TFA was mixed and heated at 95°C for 24 h. NPCN-333 was collected by centrifugation.

Synthesis of FNPCN-333

30 mg NPCN-333 was dispersed in 5 mL DMF in which was added 5 mL 10 mg/mL DMF solution of BTB-Green. The mixture was kept in 85°C oven for 4 h and the solid was collected by centrifugation. The determination of the amount of metathesized ligand was conducted by digesting the obtained solid in HCl, dried under vacuum and dissolved in deuterated DMSO for NMR analysis. The ligand ratio of BTB-Green/TATB is 1:6.

Stepwise encapsulation of SOD and CAT on FNPCN-333

Stock solutions of SOD (5 mg/mL) and CAT (5 mg/mL) were prepared by dissolving SOD and CAT in deionized water, respectively. 1 mg FNPCN-333 was suspended in water in which 1 mL CAT stock solution was added. The mixture was vortexed for 20 minutes and the solid was collected by centrifugation and washed by fresh water for 3 times. The solid was re-suspended in 1 mL water and 1 mL SOD stock solution was added. The mixture was kept vortexing for 20 minutes and the solid was collected by centrifugation. SC@FNPCN-333 was washed with fresh water for 3 times before re-suspended in 0.3 mL water.

WST assay for determining SOD activity

WST assay kit is purchased from Sigma Aldrich. 20 µL sample solution containing different concentrations of SC@FNPCN-333 is mixed with 200 µL working solution. Then 20 µL WST solution (1mL WST stock solution diluted by 19 mL working solution) is added and well mixed. Finally 20 µL xanthine oxidase (XOD) solution (15 µL XOD stock solution diluted with 2.5 mL working solution) is added and the solution is incubated at 37°C for 30 minutes. The reading at 450 nm is collected by UV-vis spectroscopy.

Amplex Red-HRP assay for determining CAT activity

20 µL sample solution containing different concentrations of SC@FNPCN-333 is mixed with 200 µL hydrogen peroxide PBS solution (final concentration is 40 µM) and incubates at 37°C for 30 minutes. Then 20 µL HRP solution (0.4 U/mL) and 20 µL Amplex Red solution (100 µM) is added and incubates at 37°C for another 30 minutes. Fluorescence is collected with excitation wavelength of 540 nm and emission wavelength of 590 nm.

Cell internalization of SC@FNPCN-333 and CLSM imaging

HeLa cells were seeded in an 8 well plate and allowed to adhere overnight. Then the culture media was replaced by 200 µL fresh nrL-15 media containing SC@FNPCN-333 with different concentrations at 37°C for 15-480 minutes in darkness. The cells used for CLSM imaging was cultured in 75 µg/mL SC@FNPCN-333 for 2 h. Representatively, for the CLSM imaging, media containing SC@FNPCN-333 was removed and cells were washed with fresh nrL-15 for 3 times before they were stained with Hoechst 33342 (5 µg/mL), Lyso Tracker red (10 µg/mL) and SYTOX Blue (5 µg/mL). The cells were then incubated for 5 minutes before they were imaged. Co-incubation of SC@FNPCN-333 and D-dfTAT (20 µM) was

conducted in the same manner. The only difference lied in the cell staining step. The cells were only stained with SYTOX Blue.

Cell lysis protocol

Cells are cultured in a 48 well plate and treated before the culture medium is removed. Cells are washed with fresh PBS for three times and five representative images are obtained by a confocal microscopy to count the cell number in the plate. Then the cells are digested by 200 µL concentrated nitric acid overnight. Each sample is measured three times by ICP-MS.

Intracellular Al content measurement at day 7

Cells (80-90% confluency) is cultured with 75 µg/mL SC@FNPCN-333 in nrL-15 for 2 h. Then the cells are washed with frest nrL-15 for three times and cultured with fresh DMEM. Cells are trypsinized and re-plated at day 1, 3, and 5. At day 7 the culture medium is removed and the cells are treatment with concentrated nitric acid overnight. The sample is measured three times by ICP-MS. Original Al content value: 104.1 ± 2.8 ng Al/10^5 cells, mean ± s.d.

***In cellulo* antioxidative activity evaluation of SC@FNPCN-333**

HeLa cells were seeded in a 48 well plate and allowed to adhere overnight. For the positive control groups, cell culture media was replaced by 200 µL fresh DMEM media containing PQ with concentrations from 0.5 mM to 10 mM. The cells were cultured at 37°C for 24 h, then the cells were washed with fresh PBS buffer for 3 times before charged with fresh nrL-15 buffer. The cells were stained with Hoechst 33342 (5 µg/mL) and SYTOX Green (5 µg/mL). To evaluate the antioxidative activity of SC@FNPCN-333, cells were treated with 75 µg/mL SC@FNPCN-333 for 2 h before charged with PQ solutions for 24 h. The washing and staining operations were the same as positive controls. Hoechst 33342 was excited for 500 ms and SYTOX Green was excited for 300 ms. 5 images were taken in each well. Each treatment condition was replicated for 3 wells.

Real-time ROS monitoring in living cells

Cells are cultured in a 48 well plate and are treated with SC@FNPCN-333 for 2 h in nrL-15. Then the medium is removed and the cells are washed with fresh nrL-15 for three times. Then the cells are incubated with nrL-15 containing 500 µM pyocianin and 5 µM superoxide detection dye for 30 minutes before imaged by a confocal microscope.

Theoretical estimation of enzyme loading in NPCN-333

In each unit cell of PCN-333, there are eight of A-cages (5.5 nm) and 16 of B-cages (4.2 nm). The volume of each unit cell = $(126\ \text{Å})^3 = 2.0 \times 10^{-18}\ cm^3$. The density of PCN-333(Al) = 0.23 g/cm^3. So the mass of each unit cell = $\rho \times V = 0.46 \times 10^{-18}$ g. Therefore, the total number of unit cells per gram of PCN-333(Al) is: $1/(0.46 \times 10^{-18}) = 2.2 \times 10^{18}$. And the A-cage in each gram of PCN-333(Al) = $2.2 \times 10^{18} \times 8 = 1.7 \times 10^{19} = 2.9 \times 10^{-5}$ mol. B-cage in each gram of PCN-333(Al) = $3.4 \times 10^{19} = 5.8 \times 10^{-5}$ mol. For SOD, M_W = 16.3 kDa, so the maximum loading is $16300 \times 5.8 \times 10^{-5} = 0.92$ g/g. For CAT, M_W = 64 kDa, so the maximum loading is $60000 \times 2.9 \times 10^{-5} = 1.74$ g/g.

Theoretical estimation of enzyme loading in FNPCN-333

In each unit cell of PCN-333, there are eight of A-cages (5.5 nm) and 16 of B-cages (4.2 nm). The volume of each unit cell = (126 Å)^3 = 2.0 × 10^{-18} cm^3. The density of FNPCN-333(Al) = 0.26 g/cm^3. So the mass of each unit cell = ρ × V = 0.52 × 10^{-18} g. Therefore, the total number of unit cells per gram of PCN-333(Al) is: 1/(0.52 x 10^{-18}) = 1.95 x 10^{18}. And the A-cage in each gram of PCN-333(Al) = 1.95 × 10^{18} × 8 = 1.5 × 10^{19} = 2.6 × 10^{-5} mol. B-cage in each gram of PCN-333(Al) = 3.0 × 10^{19} = 5.2 × 10^{-5} mol. For SOD, M_W = 16.3 kDa, so the maximum loading is 16300 × 5.2 × 10^{-5} = 0.82 g/g. For CAT, M_W = 64 kDa, so the maximum loading is 60000 × 2.6 × 10^{-5} = 1.56 g/g.

Colocalization coefficient estimation

For cellular colocalization experiments, the Manders' overlap coefficient R (measures how interdependent the red and green channels are) and colocalization coefficient M1 (measures the percentage of above-background pixels in the red channel that overlap with the above-background pixels in the green channel) were calculated using ImageJ (NIH).

Supplementary Table 1. Sizes and surface charges of NPCN-333, FNPCN-333, SC@FNPCN-333 before and after soaking in DMEM for 7 days. n=3, mean ± s.d.

Sample name	DLS size/nm	PDI	Zeta-potential/mV
NPCN-333 (DMF)	114.9 ± 31.5	0.207	18.27 ± 1.17
FNPCN-333 (DMF)	131.8 ± 36.3	0.202	1.00 ± 0.03
SC@FNPCN-333 (DMEM)	154.3 ± 36.5	0.177	1.23 ± 0.14
SC@FNPCN-333 soaked in DMEM for 7 days (DMEM)	142.2 ± 44.3	0.204	0.38 ± 0.06

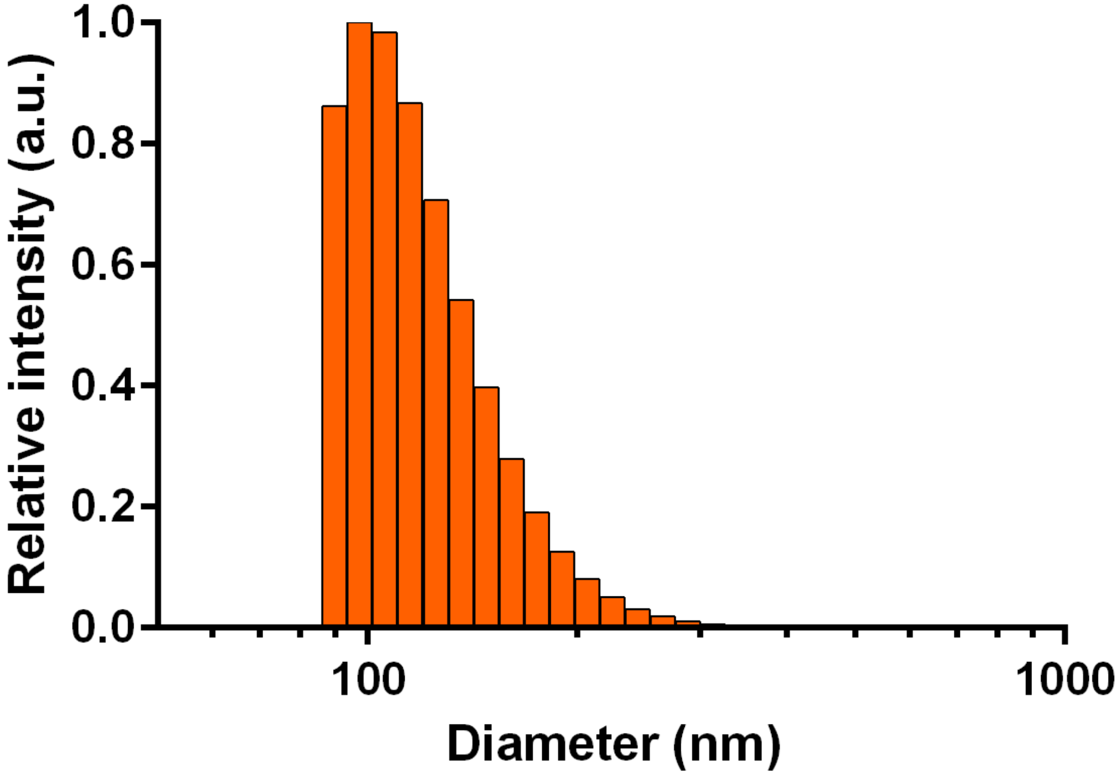

Supplementary Figure 1. DLS result of NPCN-333 suspended in DMF.

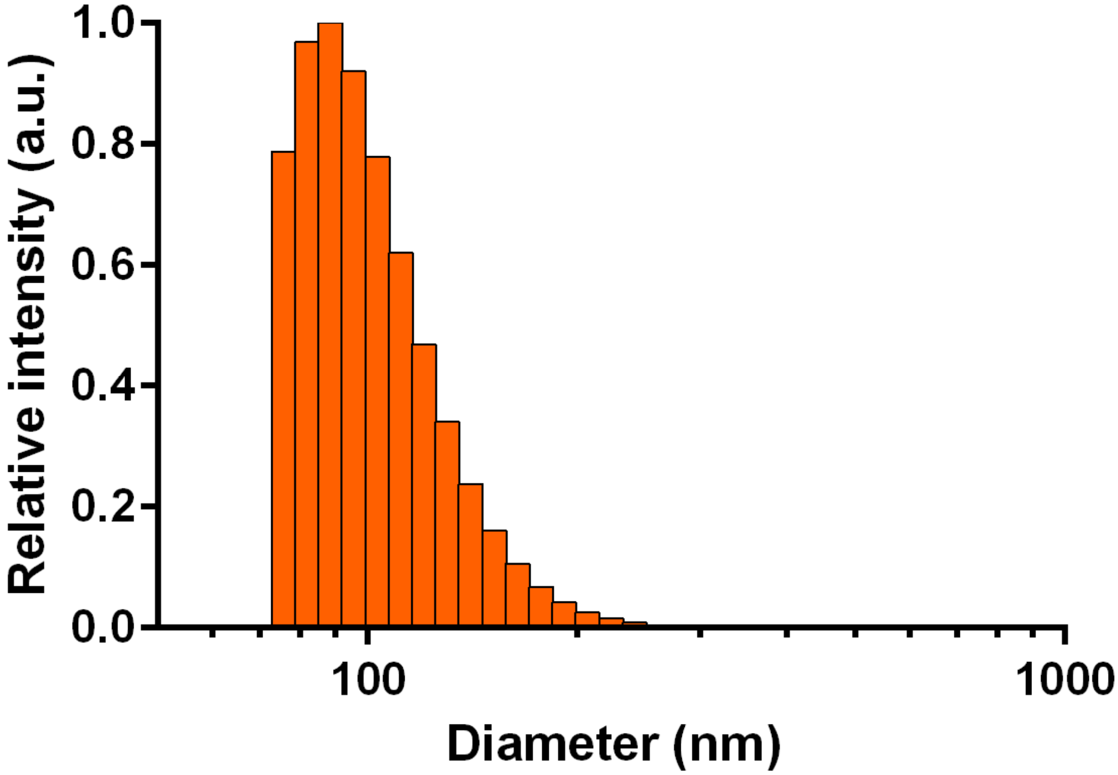

Supplementary Figure 2. DLS result of FNPCN-333 suspended in DMF.

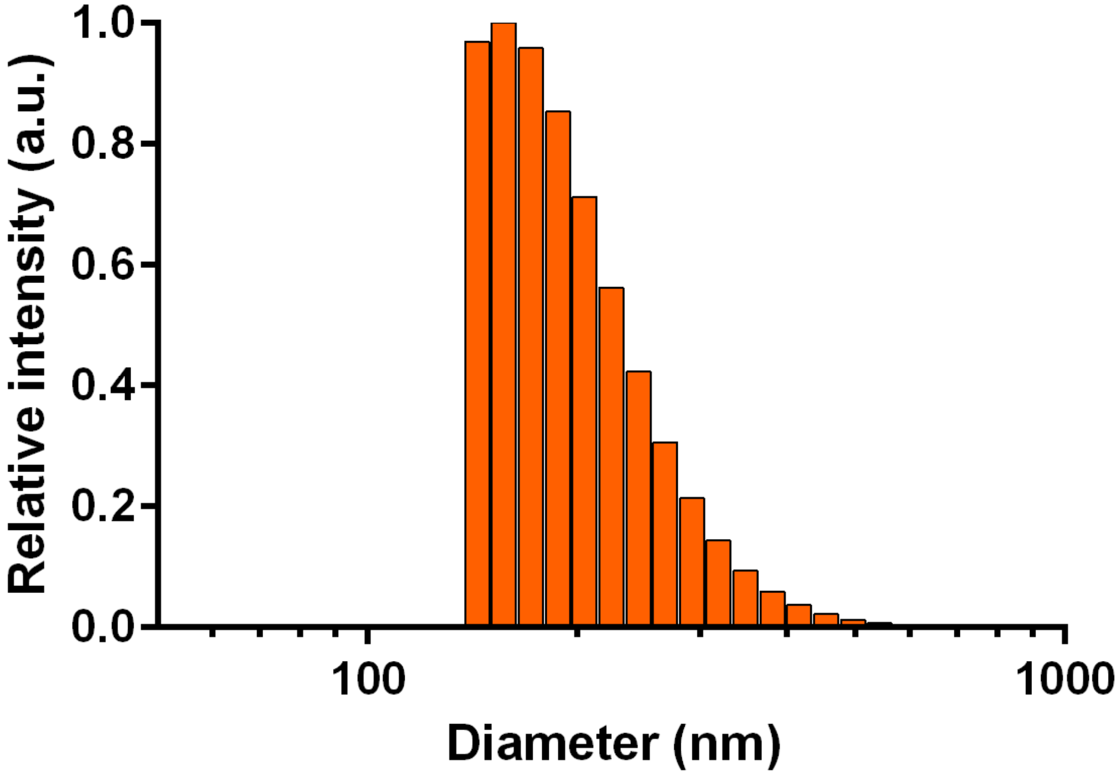

Supplementary Figure 3. DLS result of SC@FNPCN-333 suspended in deionized water.

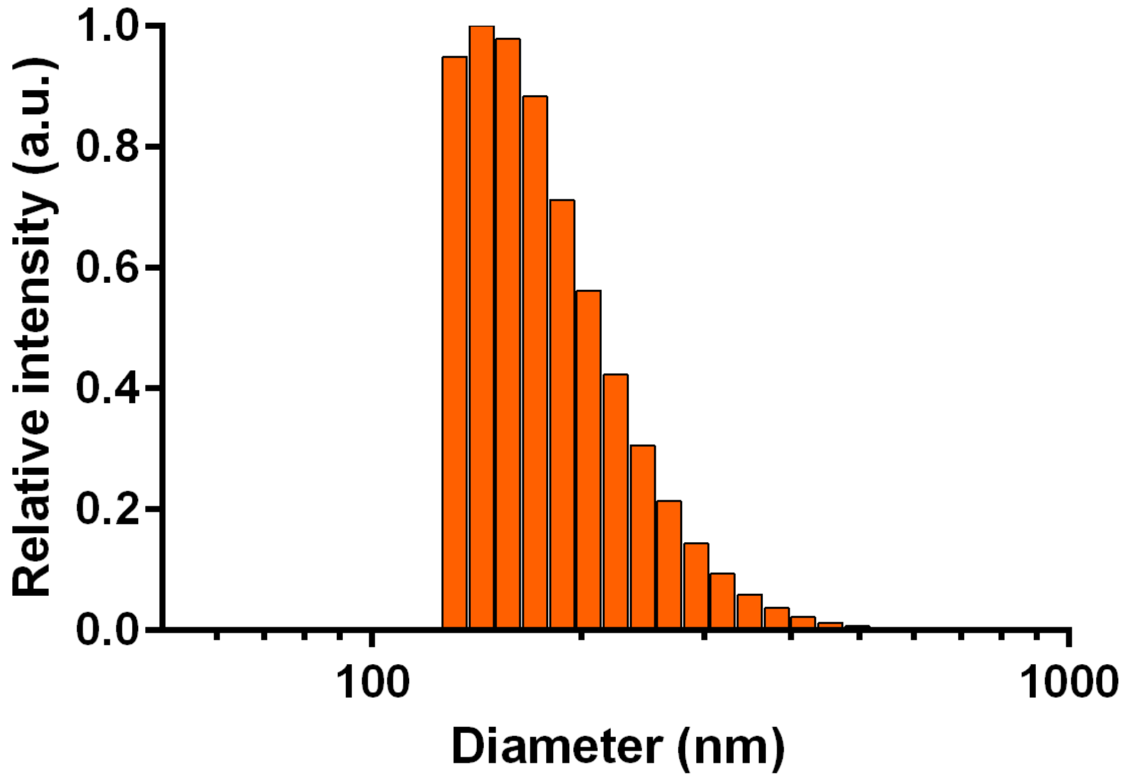

Supplementary Figure 4. DLS result of SC@FNPCN-333 after soaking in DMEM for 7 days. The sample was suspended in deionized water.

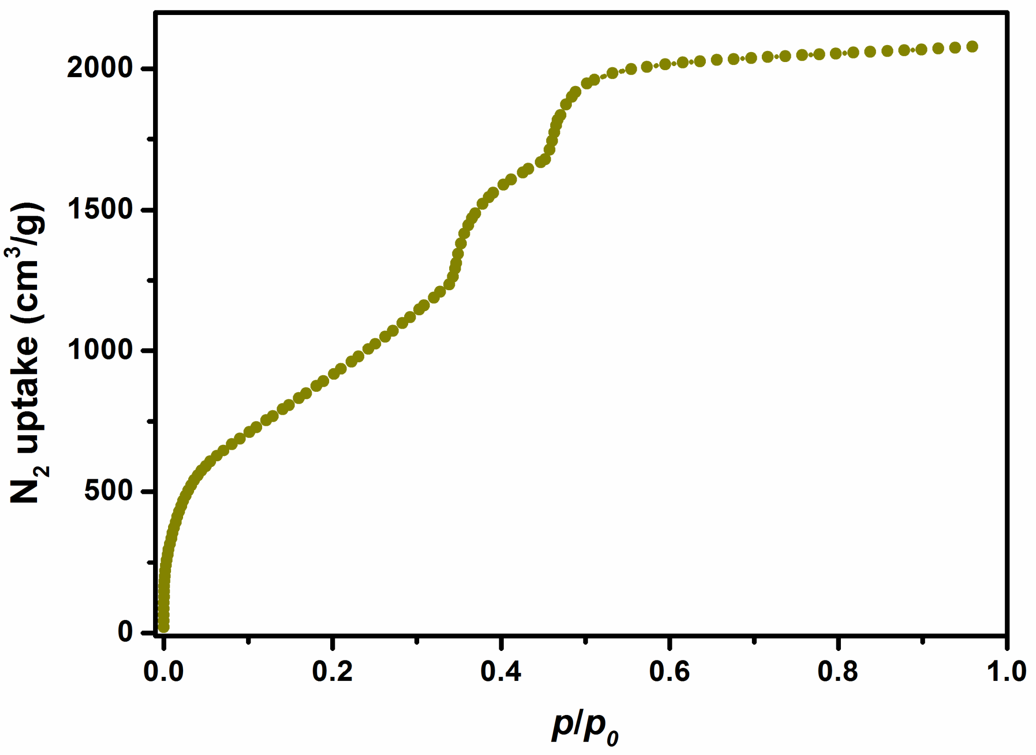

Supplementary Figure 5. N_2 isotherm of NPCN-333 at 77K.

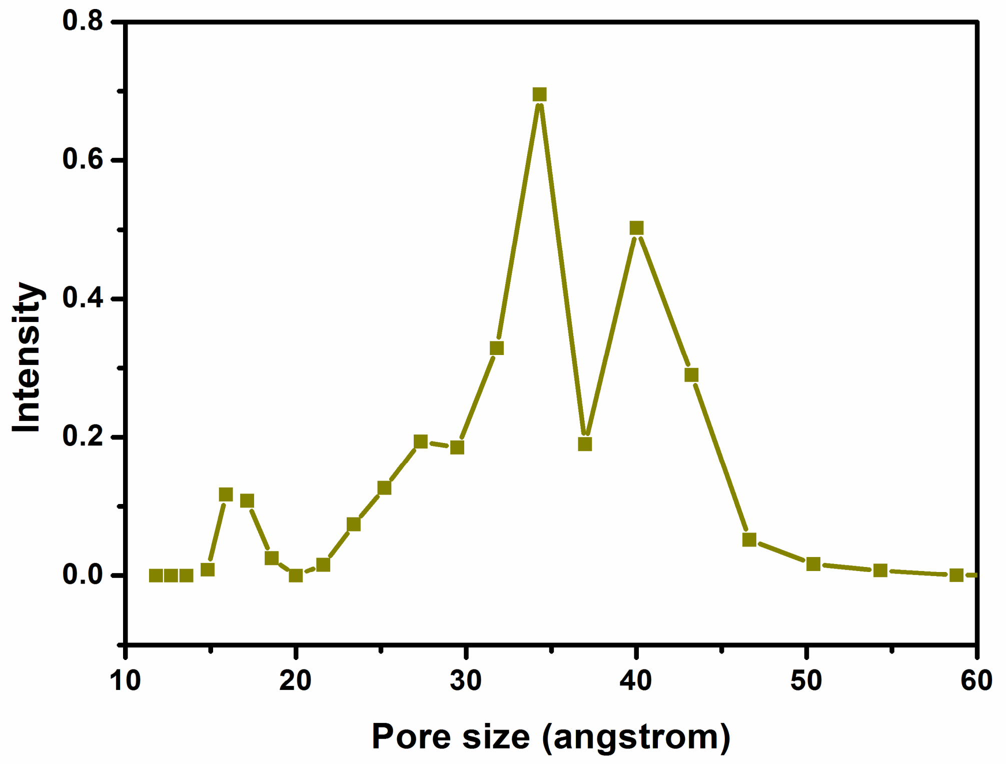

Supplementary Figure 6. Pore size distribution of NPCN-333 calculated from N_2 isotherm at 77K.

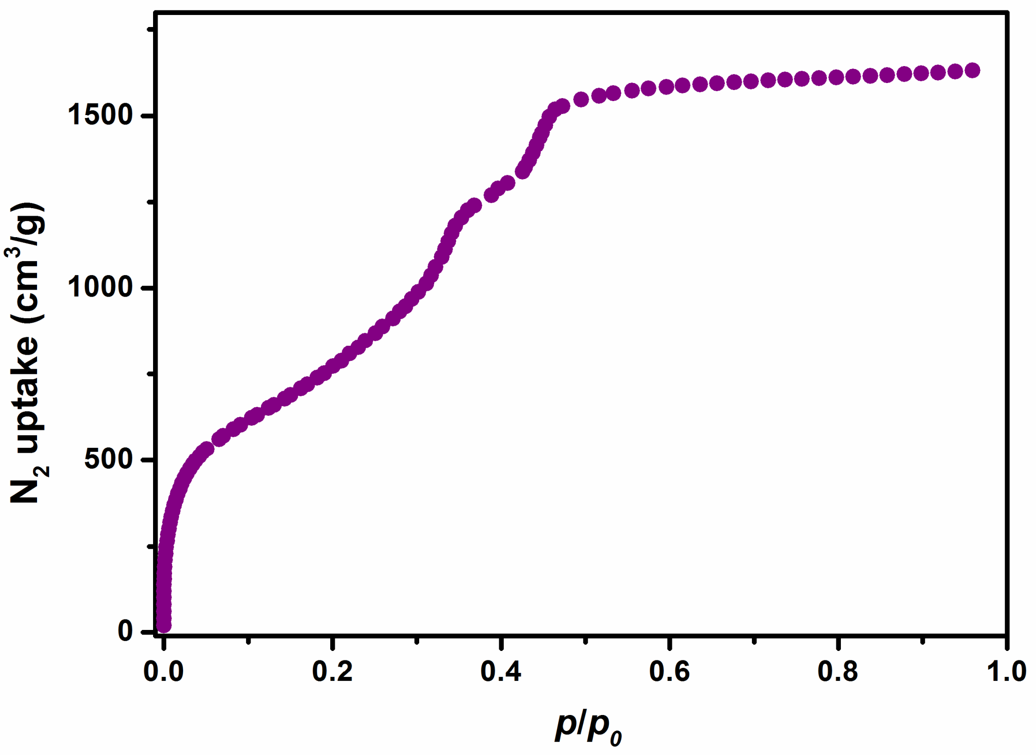

Supplementary Figure 7. N_2 isotherm of FNPCN-333 at 77K.

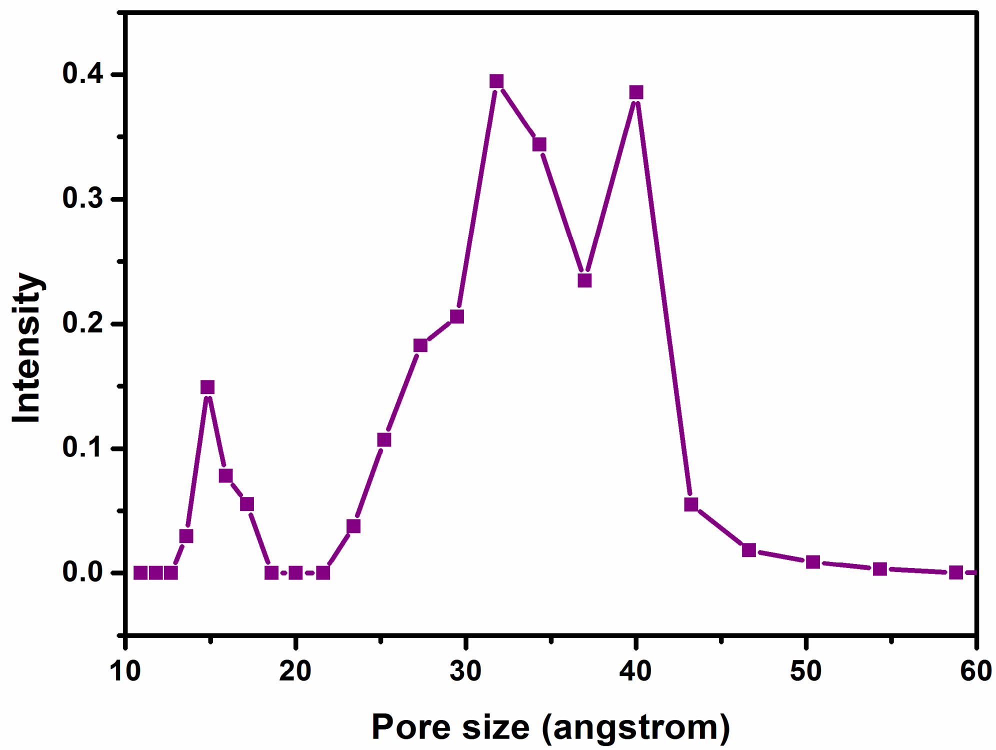

Supplementary Figure 8. Pore size distribution of FNPCN-333 calculated from N_2 isotherm at 77K.

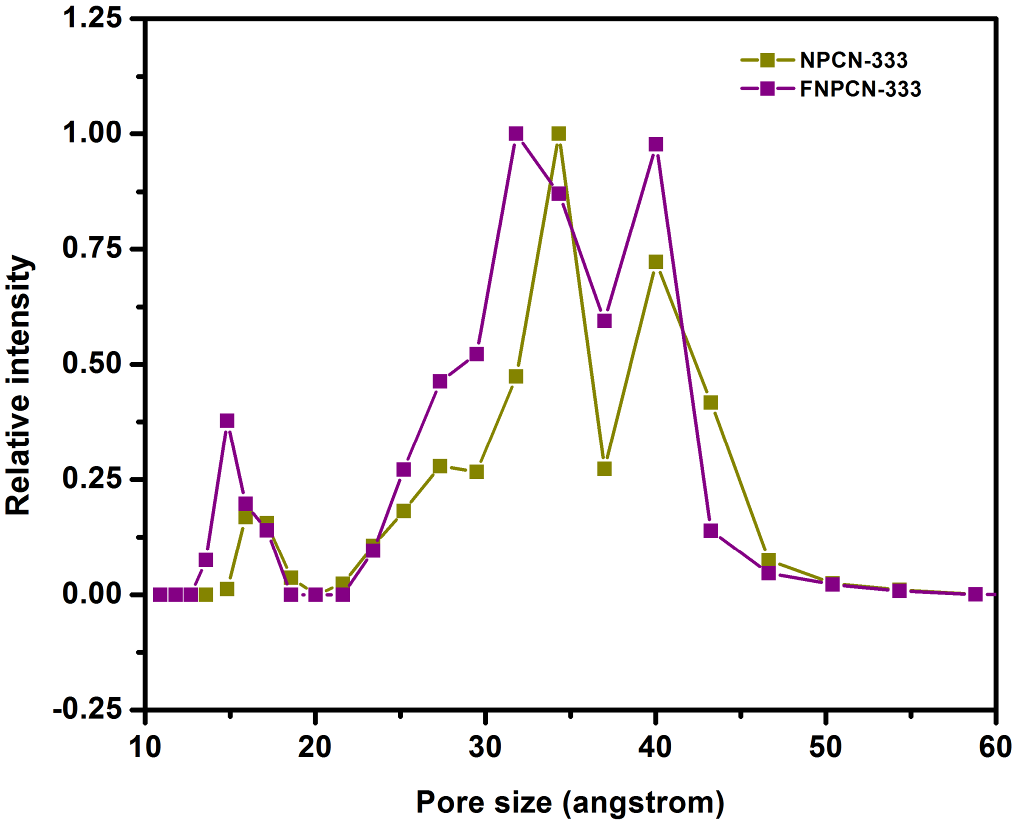

Supplementary Figure 9. Relative pore size distribution of NPCN-333 vs. FNPCN-333.

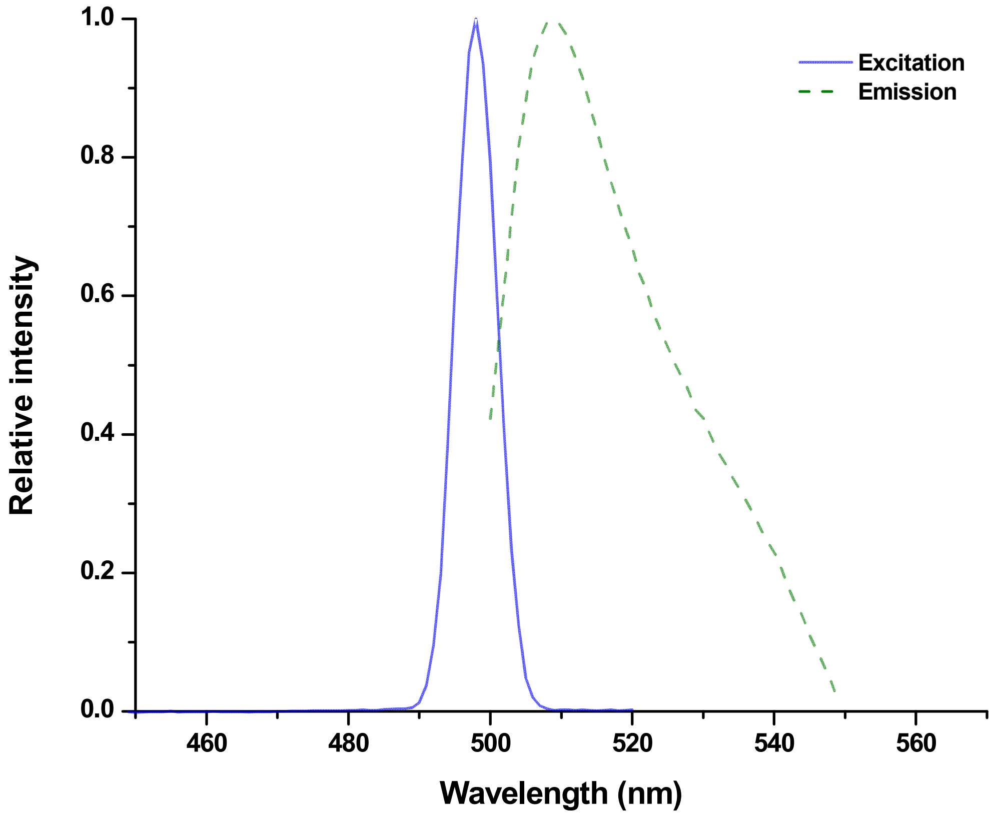

Supplementary Figure 10. Excitation (blue) and emission (green) of SC@FNPCN-333 in deionized water.

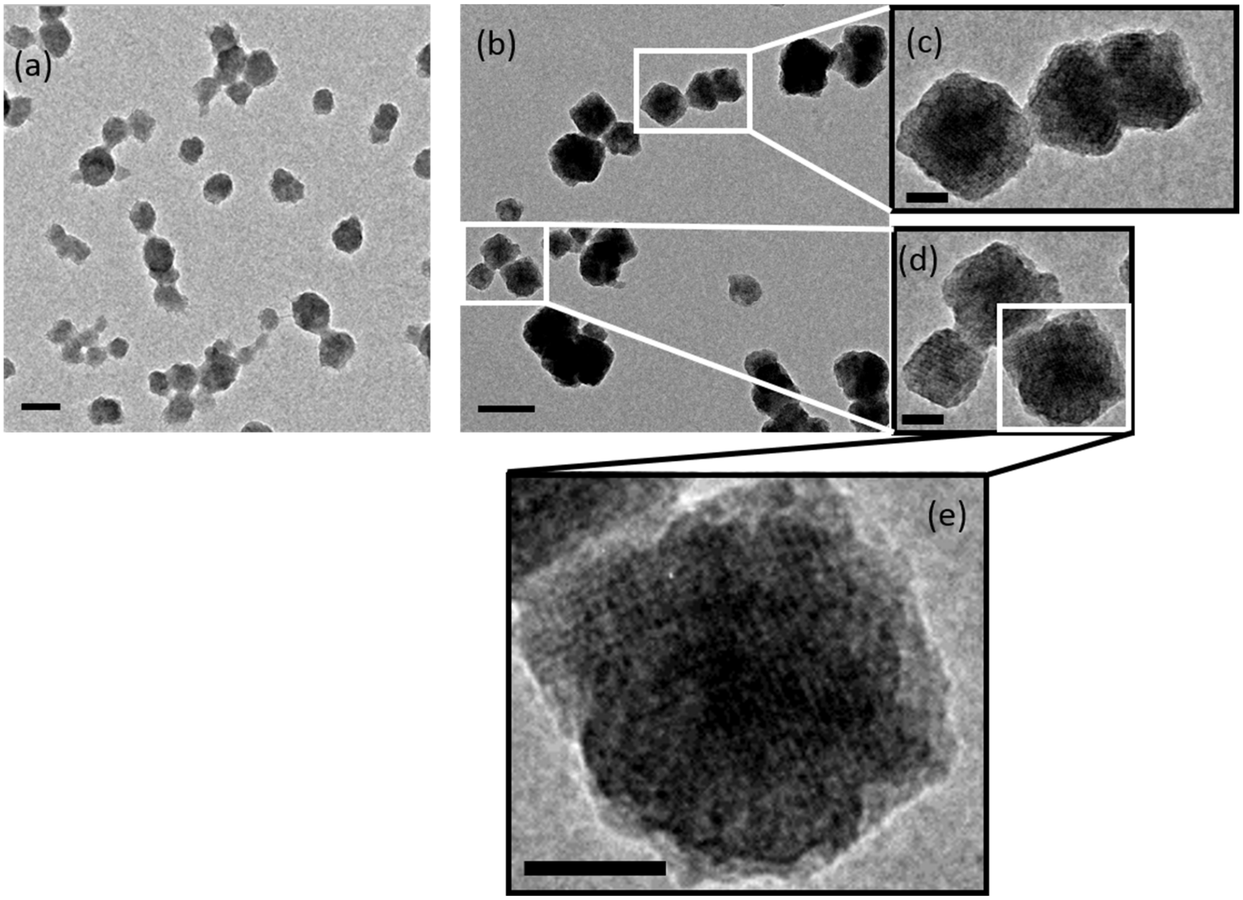

Supplementary Figure 11. TEM images of SC@FNPCN-333 (a) and NPCN-333 after soaking in DMEM for 7 days (b). The zoomed in images show the fringes on the crystals. Scale bar: 100 nm (a), 200 nm (b), 50 nm (c-e).

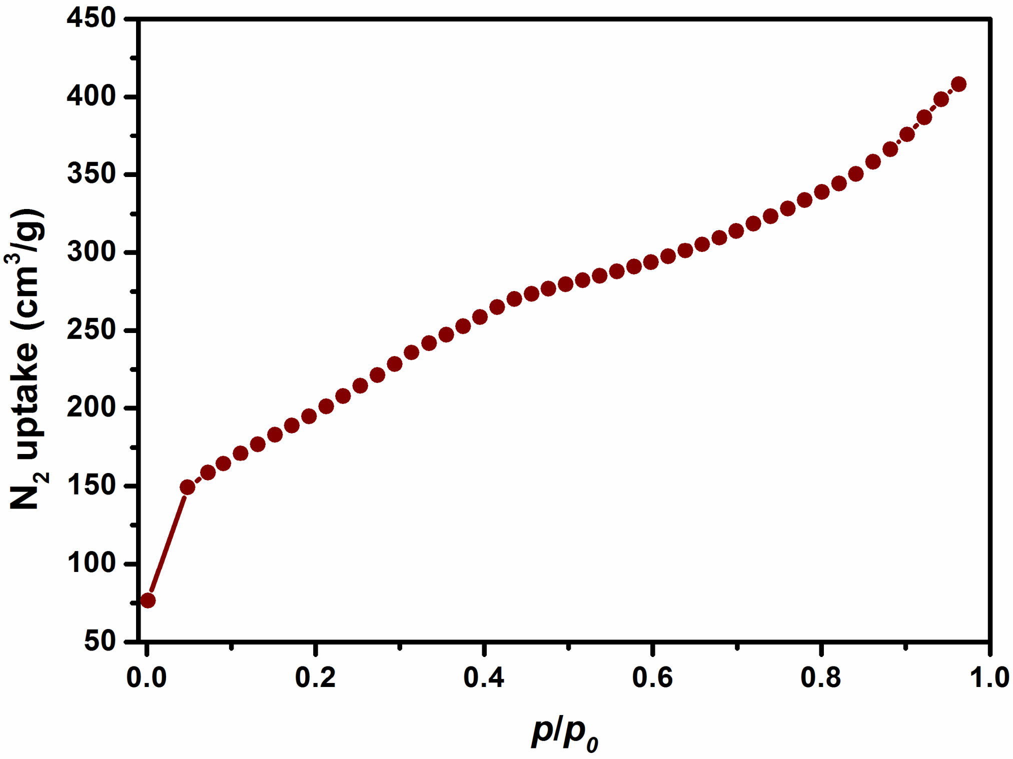

Supplementary Figure 12. N_2 isotherm of C@FNPCN-333 at 77K.

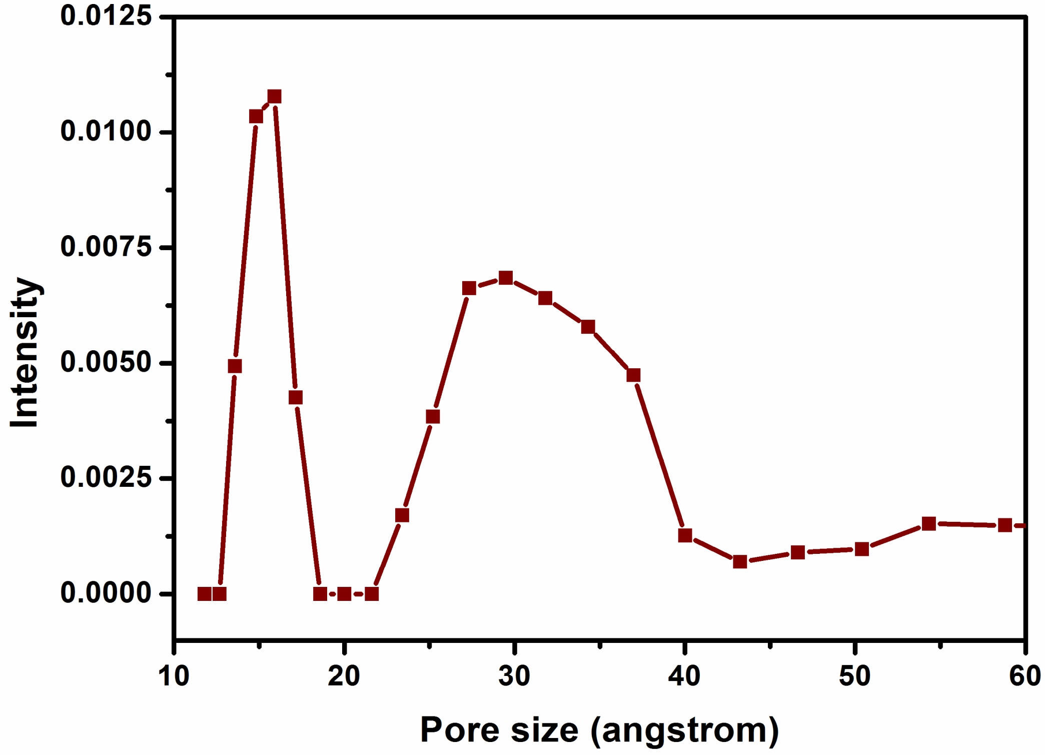

Supplementary Figure 13. Pore size distribution of C@FNPCN-333 calculated from N_2 isotherm at 77K.

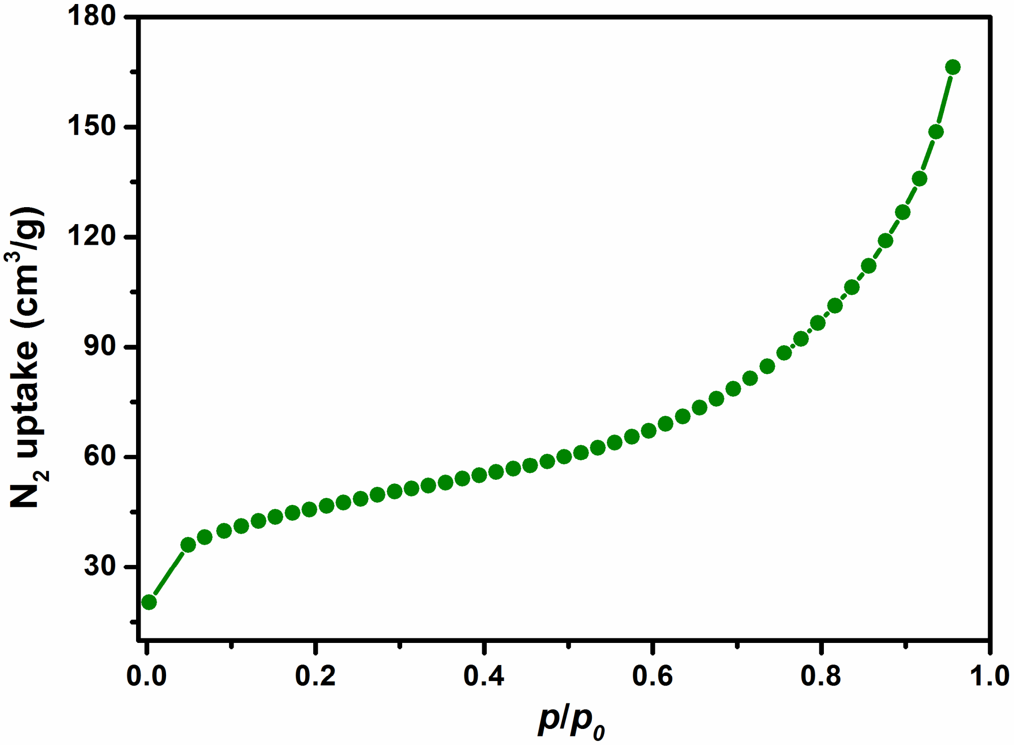

Supplementary Figure 14. N_2 isotherm of SC@FNPCN-333 at 77K.

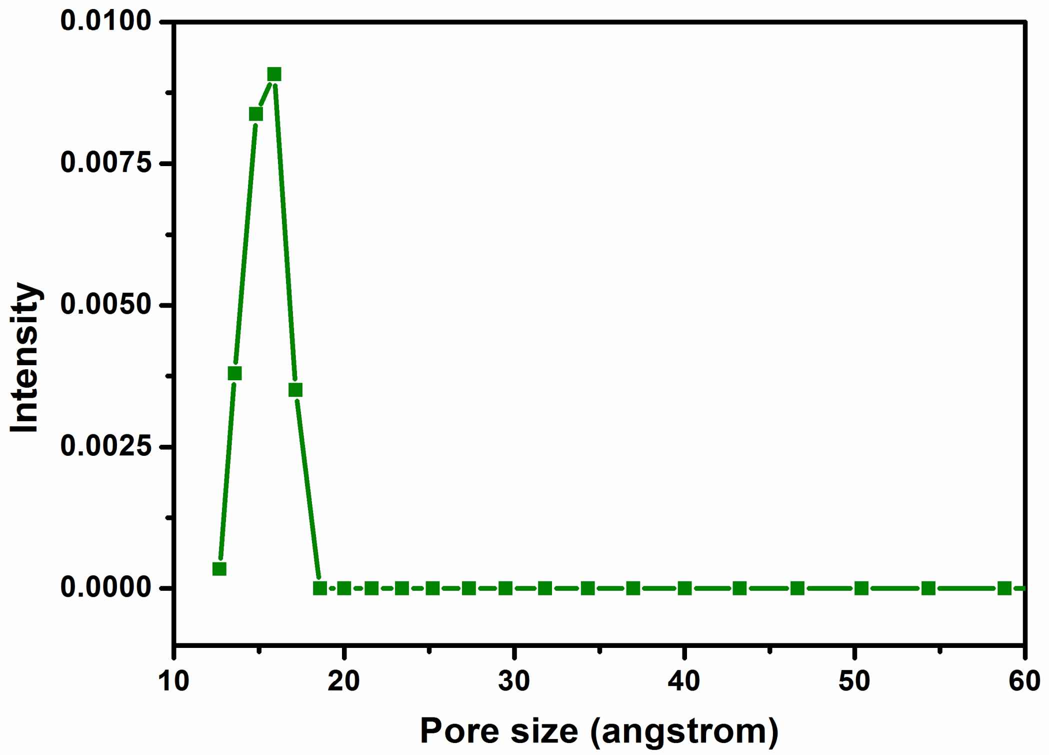

Supplementary Figure 15. Pore size distribution of SC@FNPCN-333 calculated from N_2 isotherm at 77K.

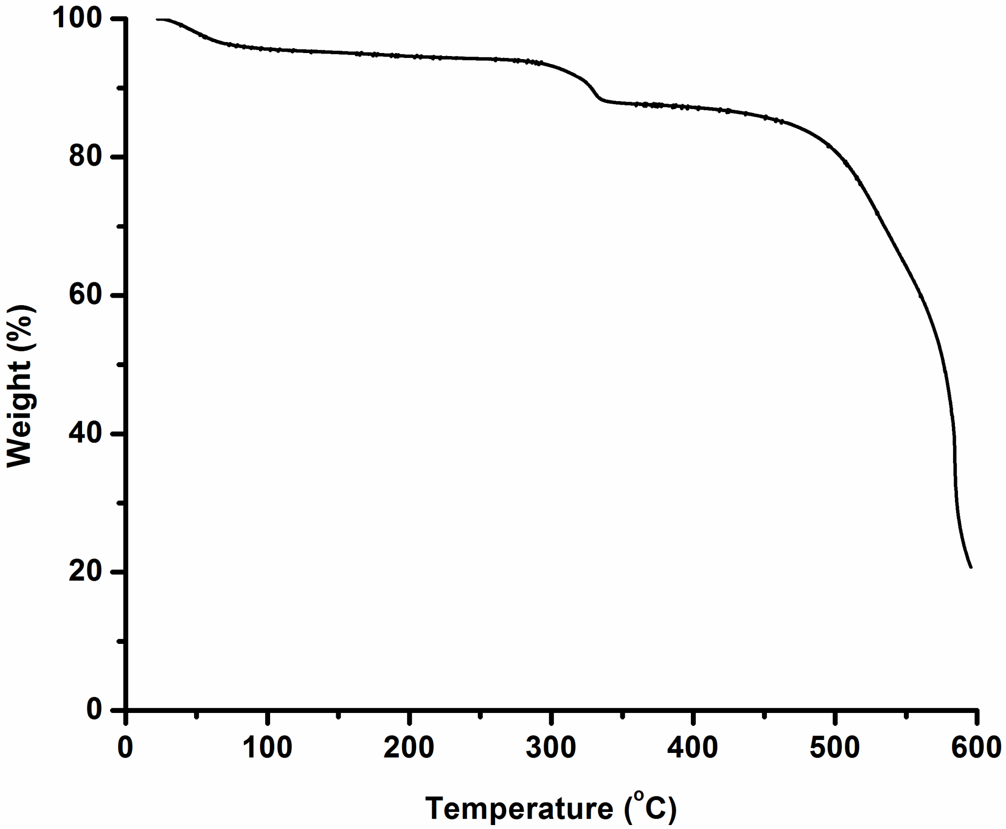

Supplementary Figure 16. TGA curve of FNPCN-333.

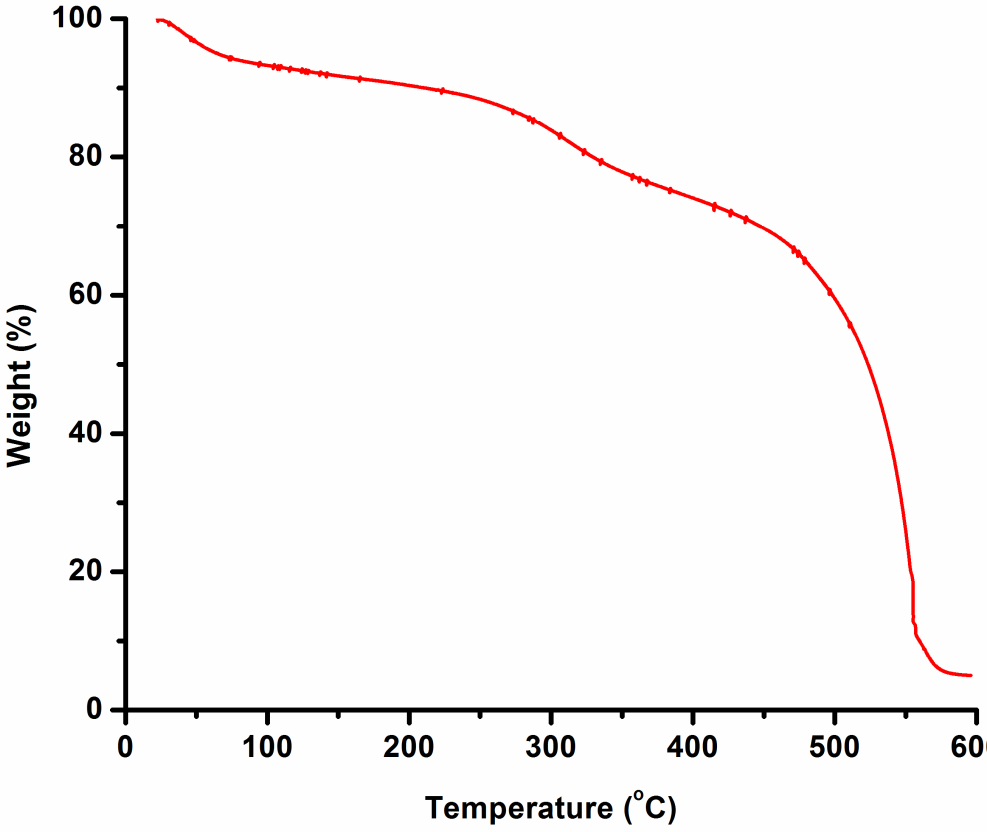

Supplementary Figure 17. TGA curve of SC@FNPCN-333.

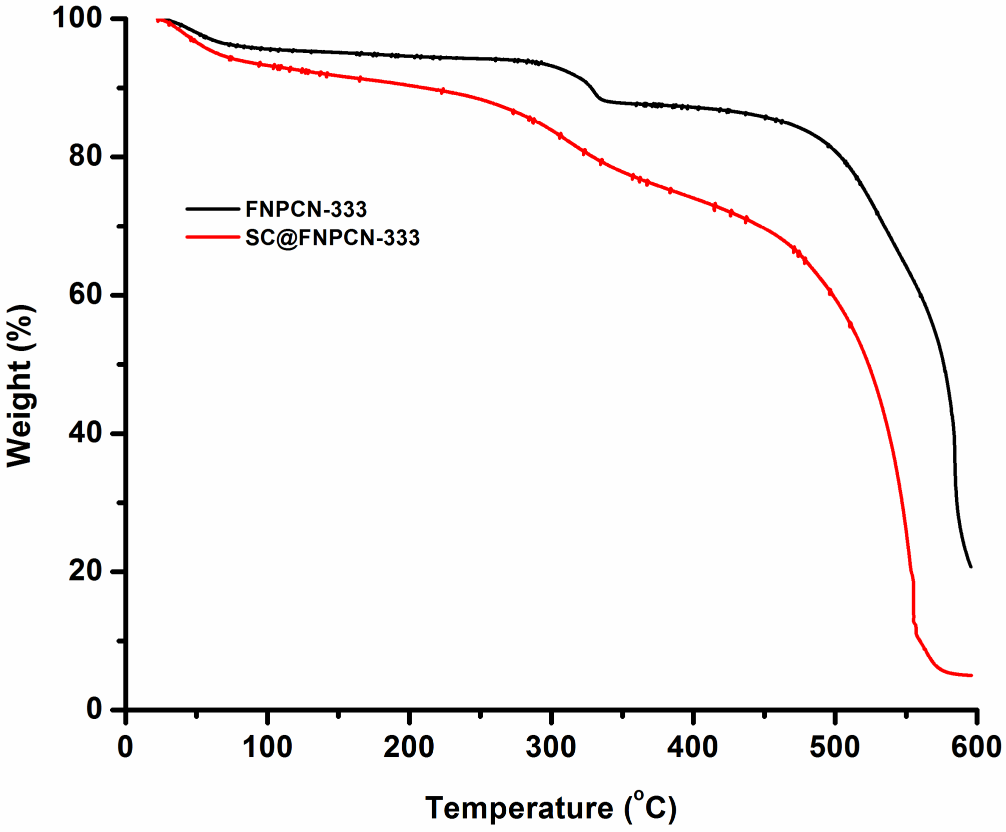

Supplementary Figure 18. Comparison of TGA curves of FNPCN-333 (black) and SC@FNPCN-333 (red). We attribute the drop in the curve of SC@FNPCN-333 at around 300 °C to the thermal decomposition of the encapsulated enzymes.

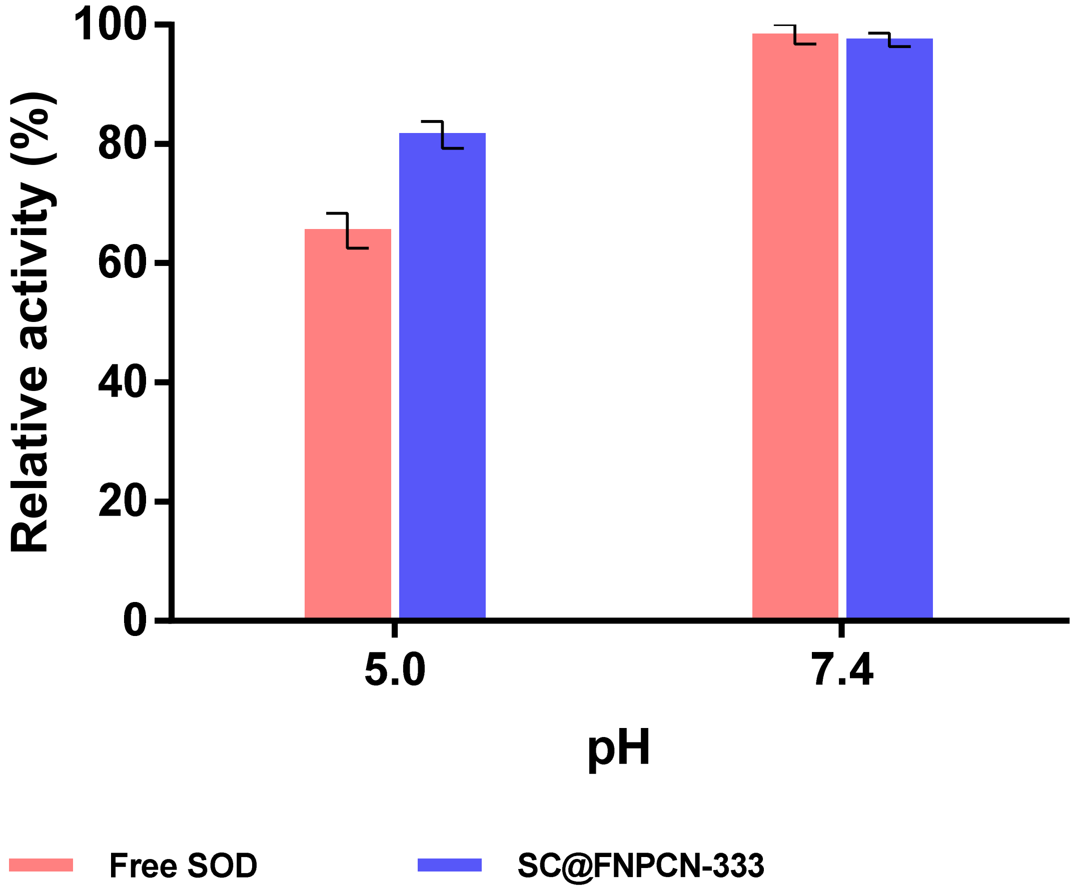

Supplementary Figure 19. Relative SOD activities of free enzyme and SC@FNPCN-333 in pH 5.0 and 7.4 buffers. n=3, mean ± s.d.

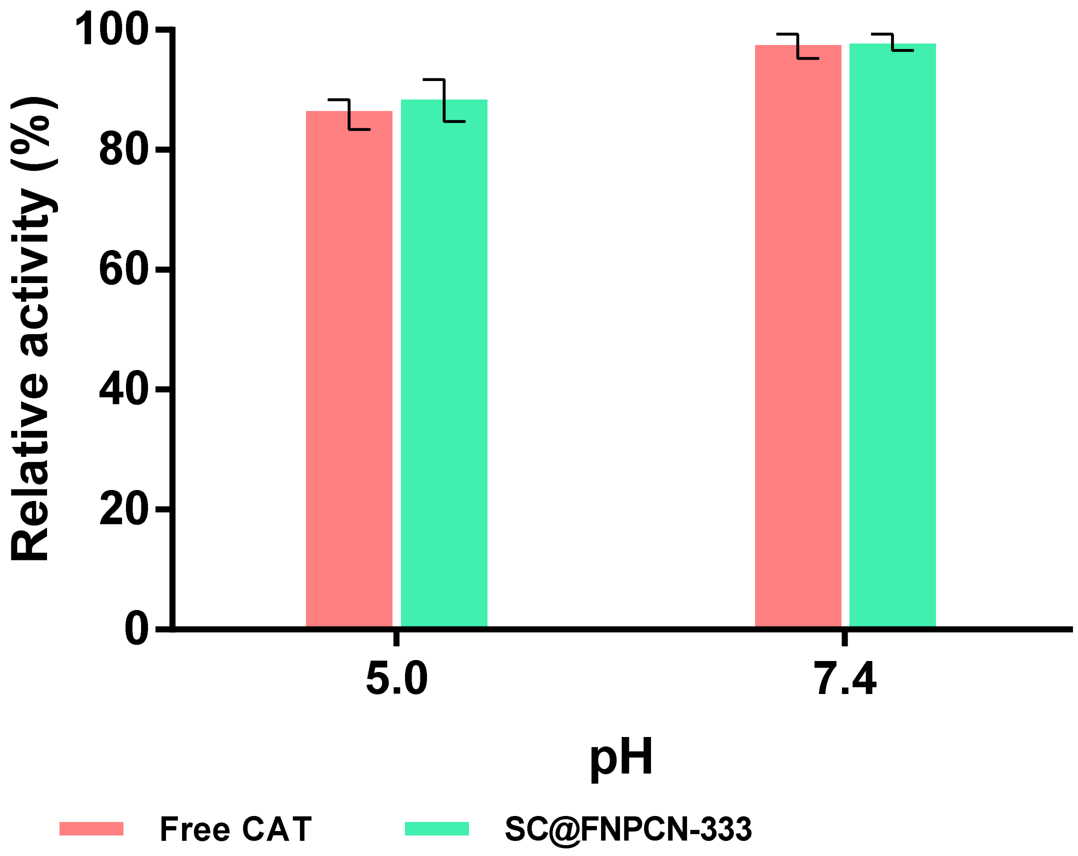

Supplementary Figure 20. Relative CAT activities of free enzyme and SC@FNPCN-333 in pH 5.0 and 7.4 buffers. n=3, mean ± s.d.

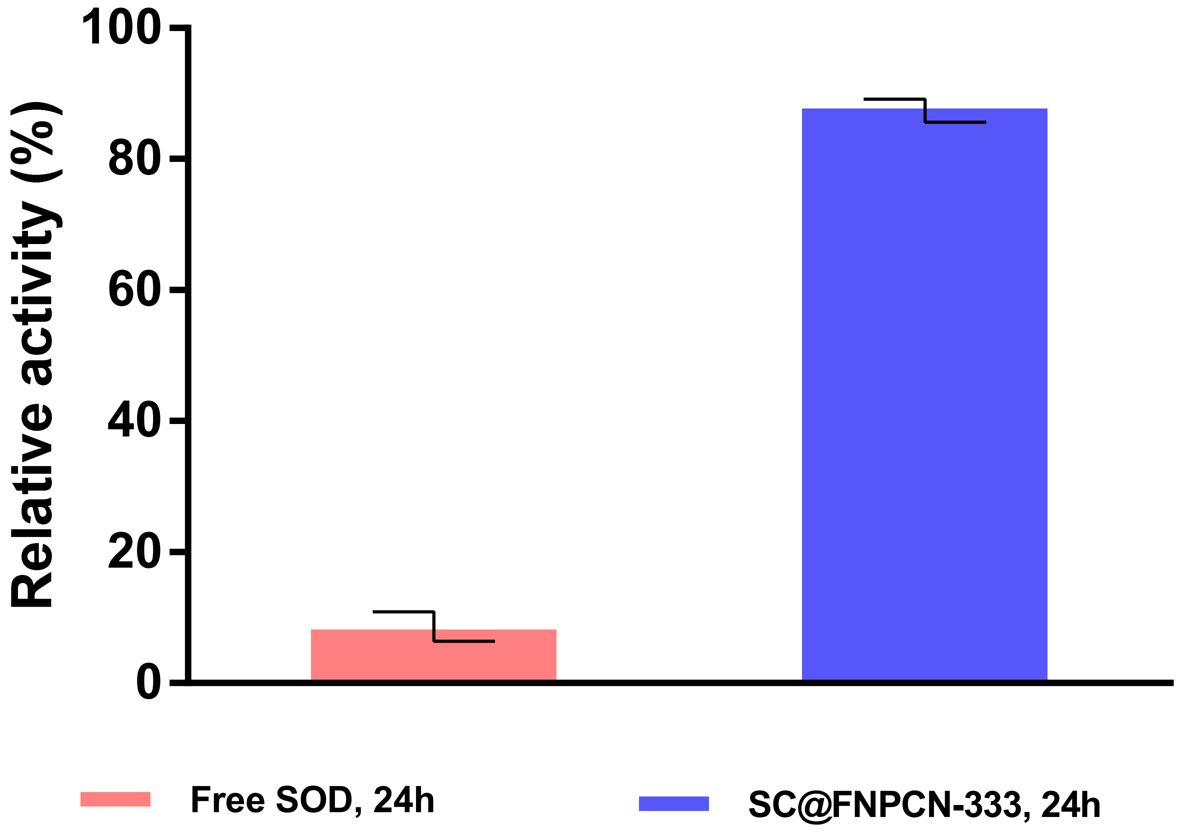

Supplementary Figure 21. Relative SOD activities of free enzyme and SC@FNPCN-333 after soaking in pH 5.0 buffer for 24 h. n=3, mean ± s.d.

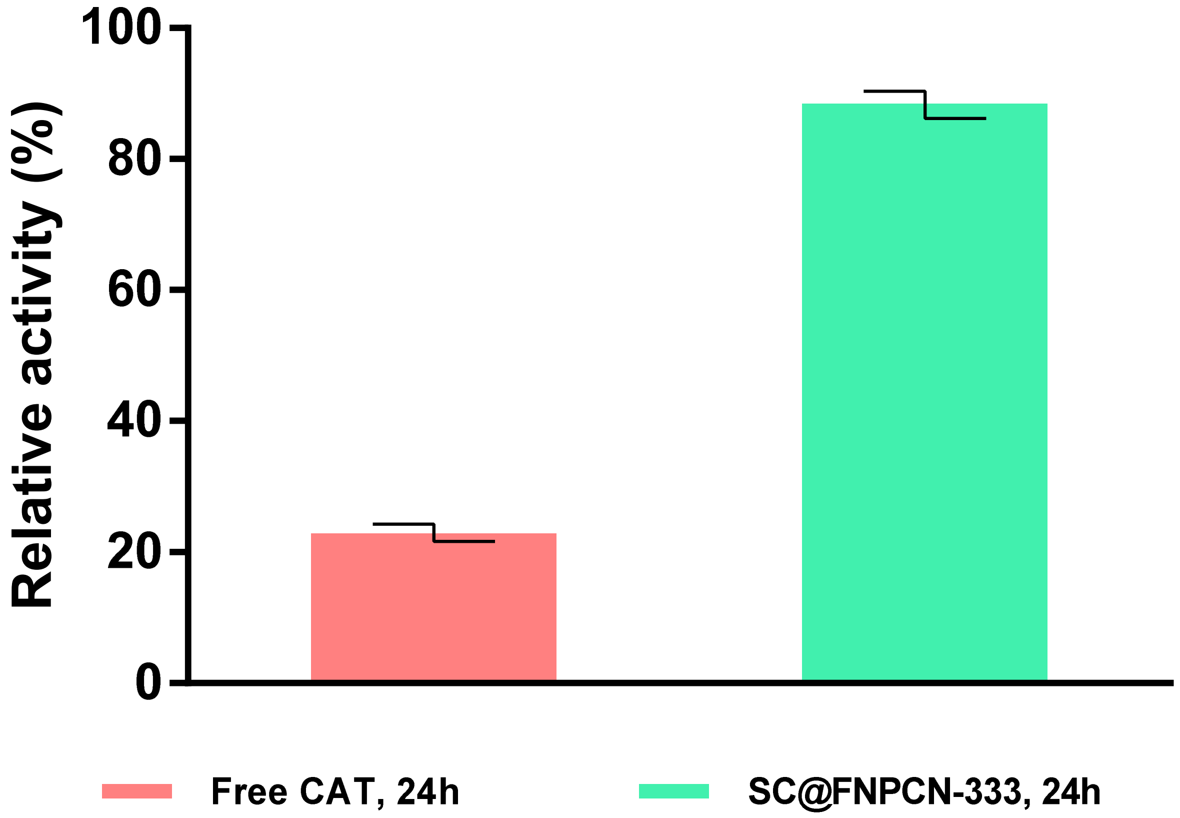

Supplementary Figure 22. Relative CAT activities of free enzyme and SC@FNPCN-333 after soaking in pH 5.0 buffer for 24 h. n=3, mean ± s.d.

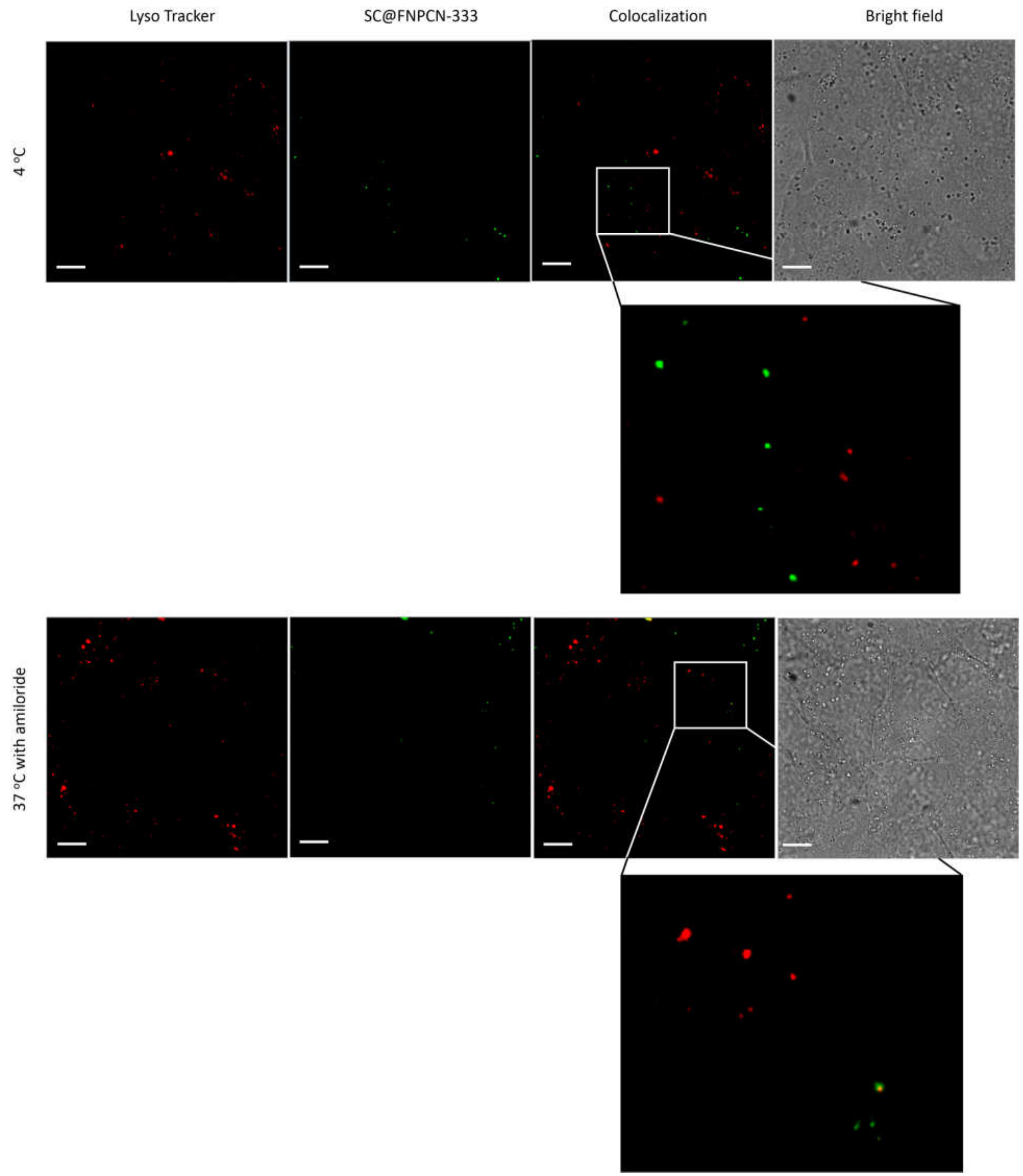

Supplementary Figure 23. Effect of low temperature (4 °C) and endocytosis inhibitor (amiloride) on the cellular internalization of SC@FNPCN-333 by HeLa cells. Zoom-in images are provided. 4 °C colocalization coefficient: 0.34 (Pearson's), 0.30 (Mander's). 37°C with amiloride colocalization coefficient: 0.61 (Pearson's), 0.58 (Mander's). Scale bar: 10 μm.

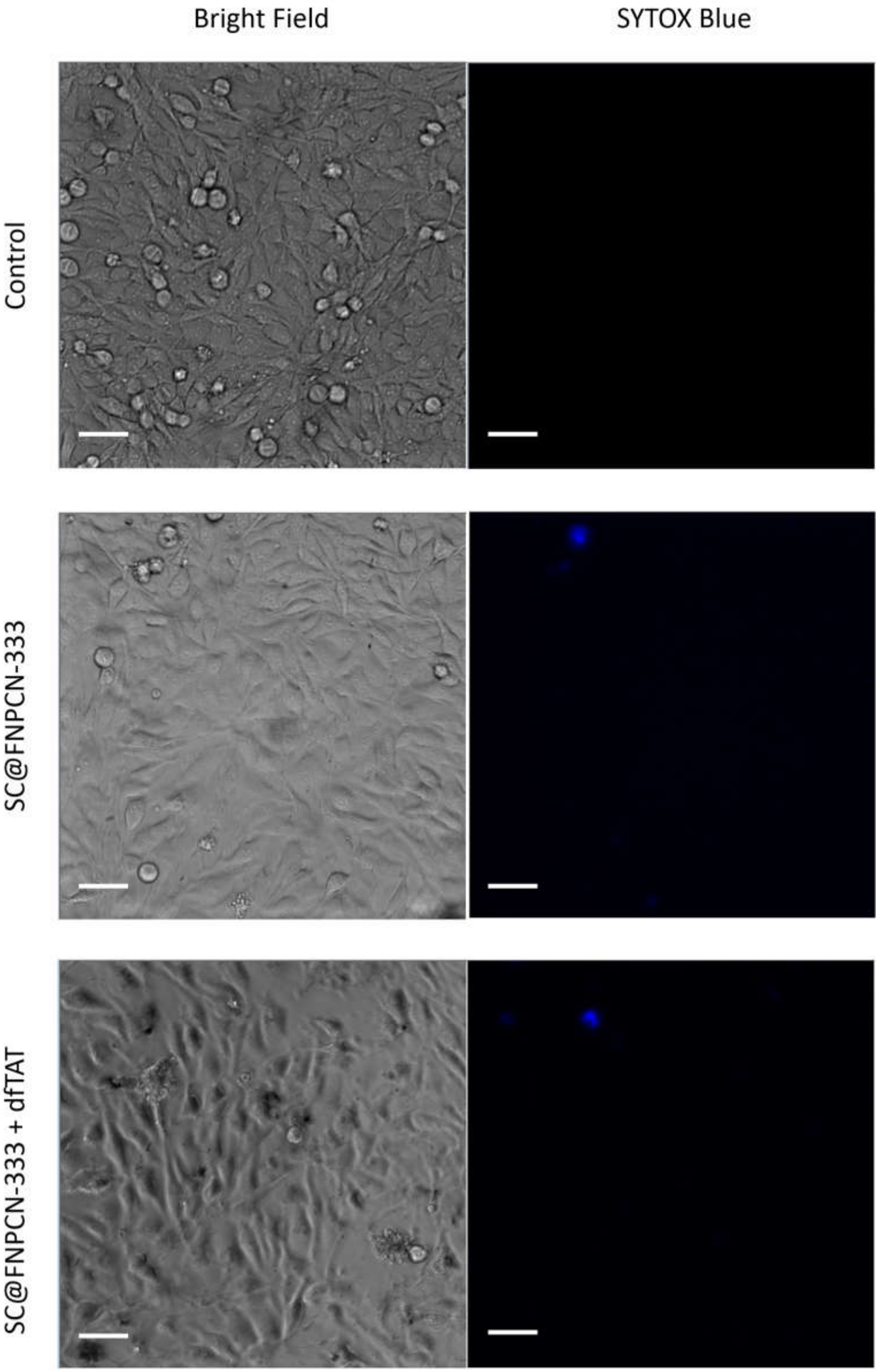

Supplementary Figure 24. SYTOX Blue assay of HeLa cells after 2 h incubation with 75 μg/mL SC@FNPCN-333 and 75 μg/mL SC@FNPCN-333 plus 20 μM D-dfTAT. Scale bar: 100 μm.

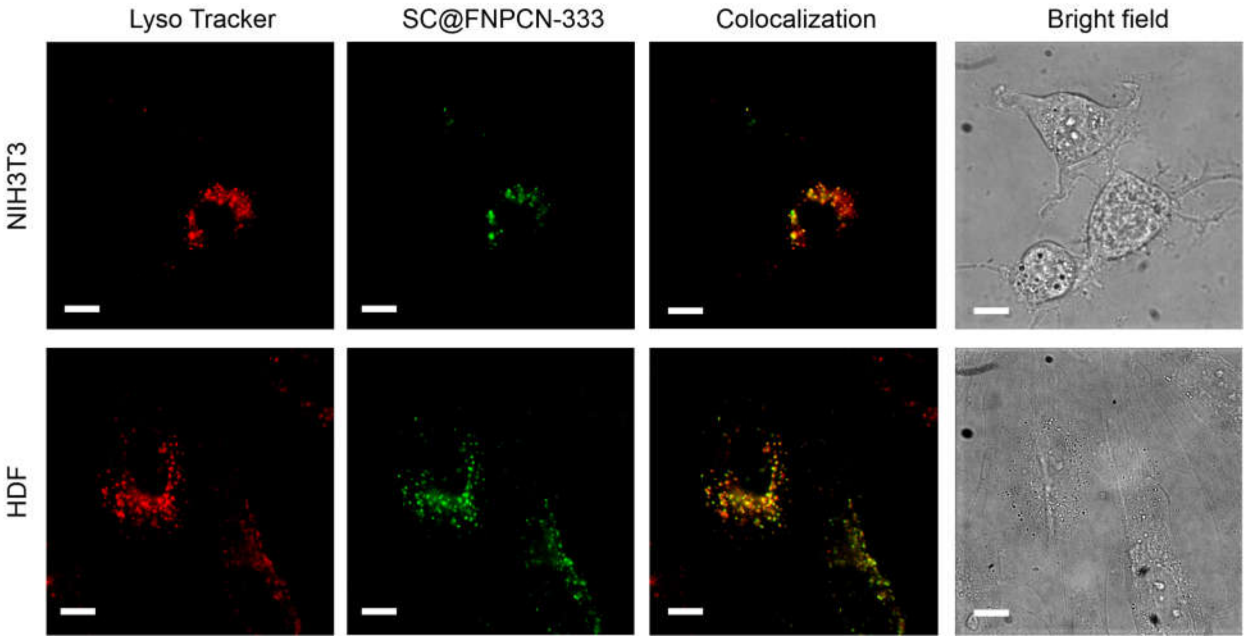

Supplementary Figure 25. SC@FNPCN-333 endocytosed by NIH3T3 and HDF cells. Colocalization coefficient: NIH3T3 0.88 (Pearson's), 0.98 (Mander's); HDF 0.94 (Pearson's), 0.99 (Mander's).

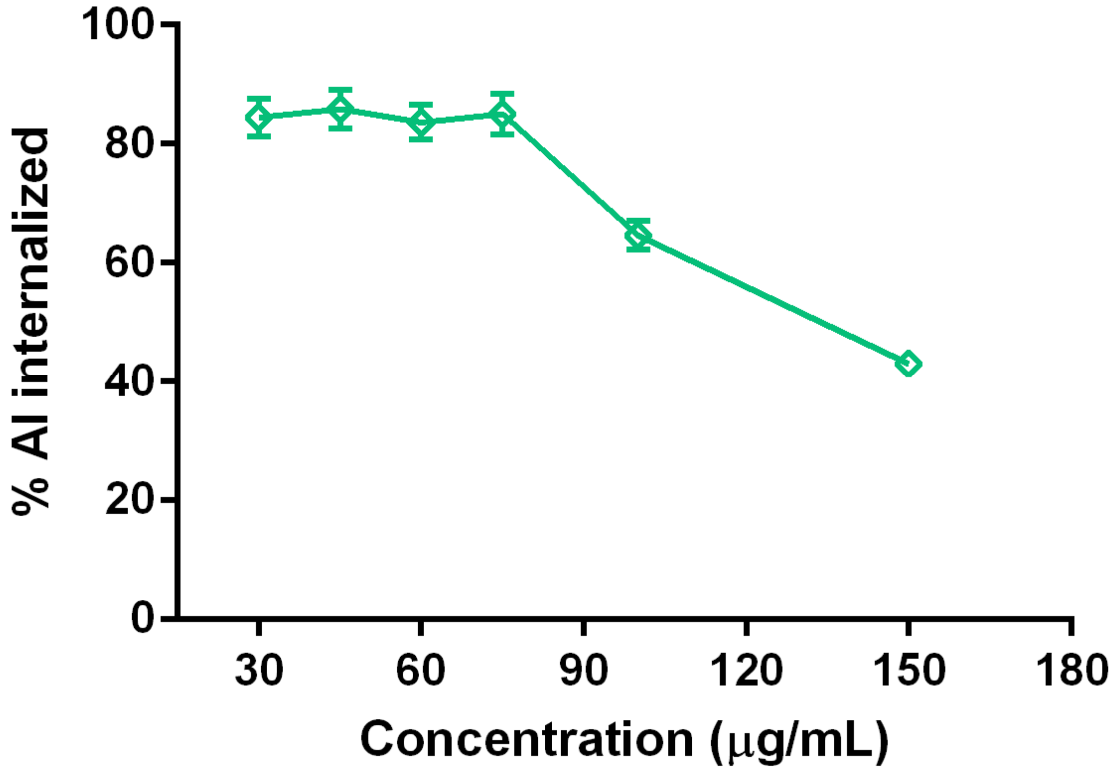

Supplementary Figure 26. Endocytosis efficiency of SC@FNPCN-333 at different nanoparticle concentration. Presented by Al % based on the ICP result and the administration dosage. n=3, mean ± s.d.

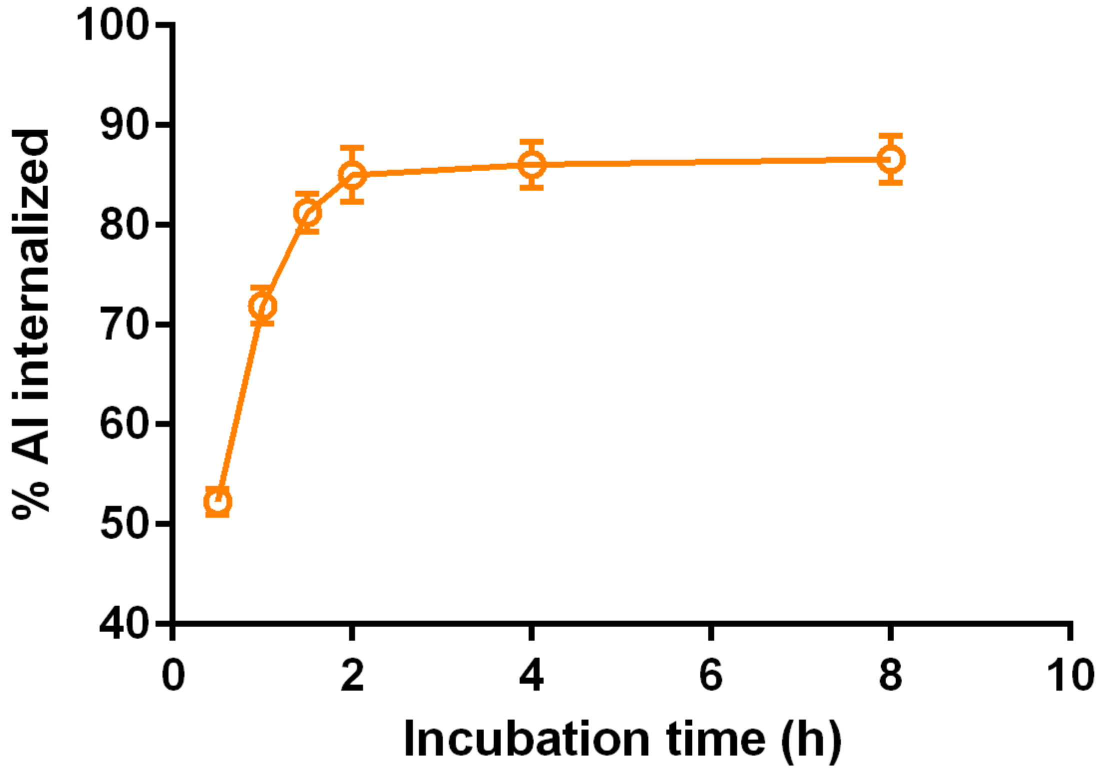

Supplementary Figure 27. Endocytosis efficiency of SC@FNPCN-333 (75 μg/mL) at different treatment time point. Presented by Al % based on the ICP result and the administration dosage. n=3, mean ± s.d.

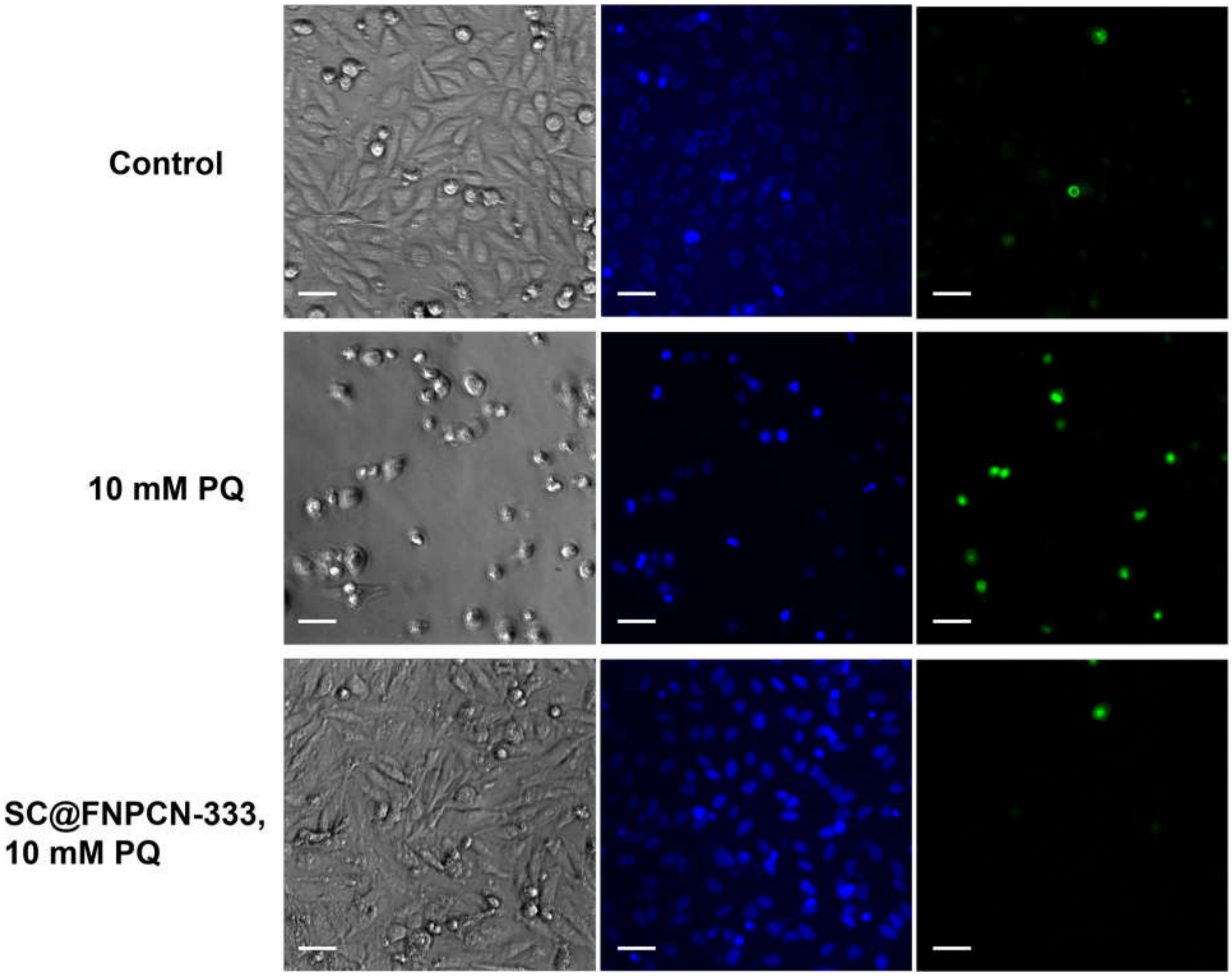

Supplementary Figure 28. CLSM images of HeLa cells as control, treated with 10 mM PQ for 24 h, pre-treated with SC@FNPCN-333 then treated with 10 mM PQ for 24 h. Left panel: bright field images; middle panel: cell nucleus stained with Hoechst 33342; right panel: dead cell nucleus stained with SYTOX Green. Scale bar: 100 µm.

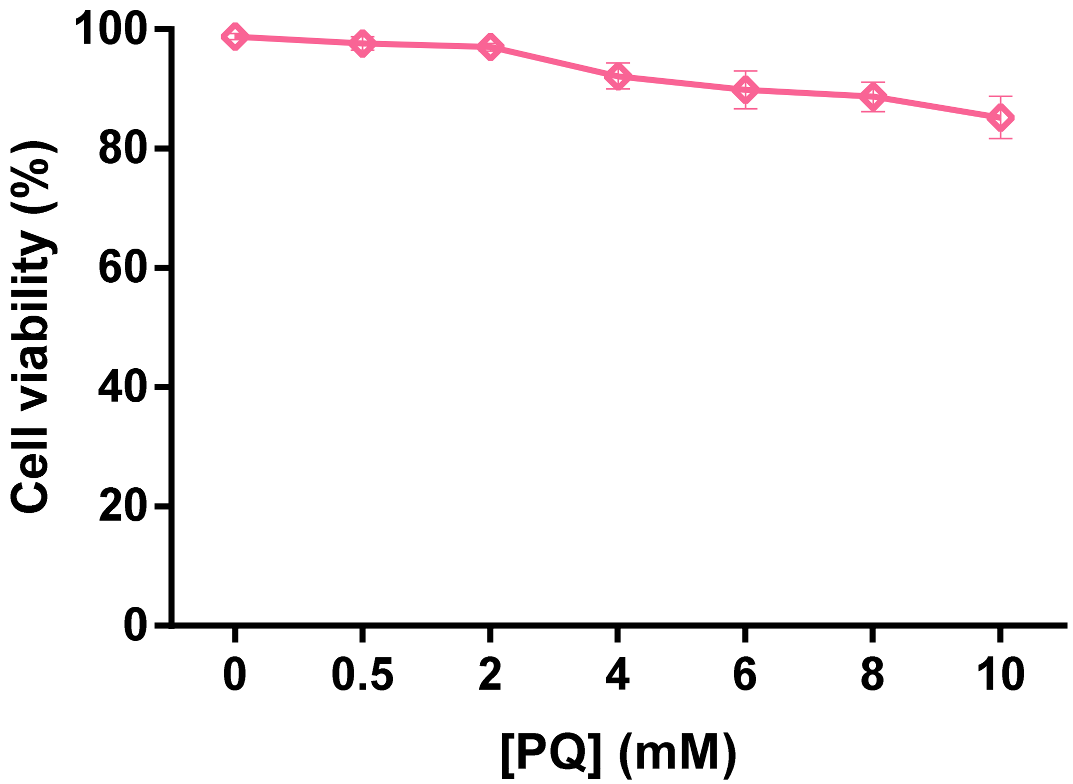

Supplementary Figure 29. Cell viability after treated with PQ for 24 h. Cells were pre-treated with 75 μg/mL SC@FNPCN-333 and 20 μM D-dfTAT for 2 h. n=5, mean ± s.d.

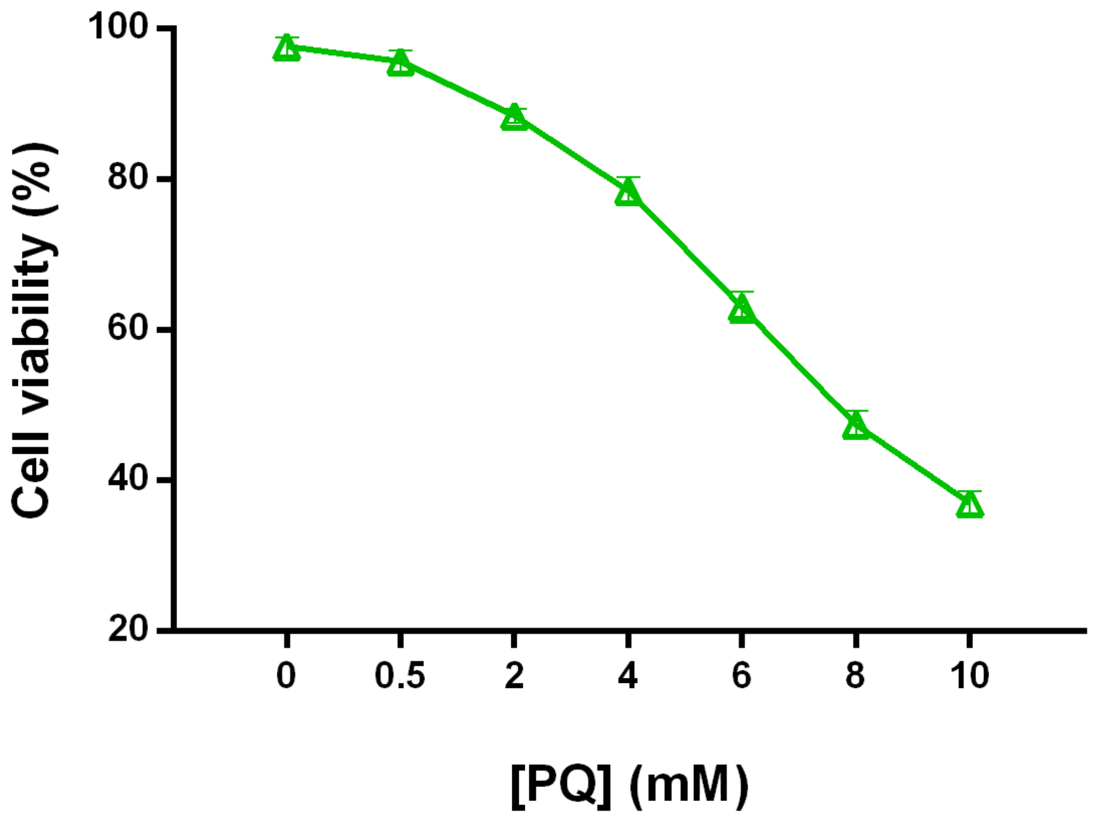

Supplementary Figure 30. Cell viability after treated with PQ for 24 h. Cells were pre-treated with 75 μg/mL S@FNPCN-333 for 2 h. n=5, mean ± s.d.

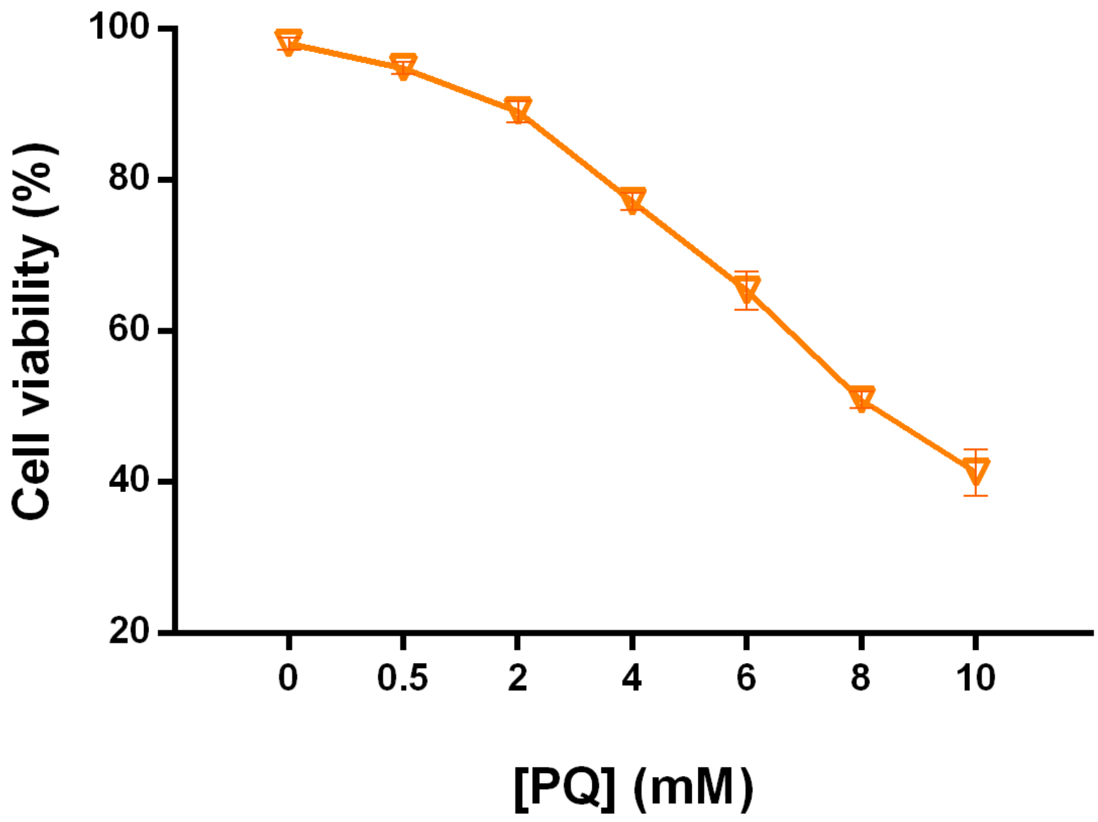

Supplementary Figure 31. Cell viability after treated with PQ for 24 h. Cells were pre-treated with 75 μg/mL C@FNPCN-333 for 2 h. n=5, mean ± s.d.

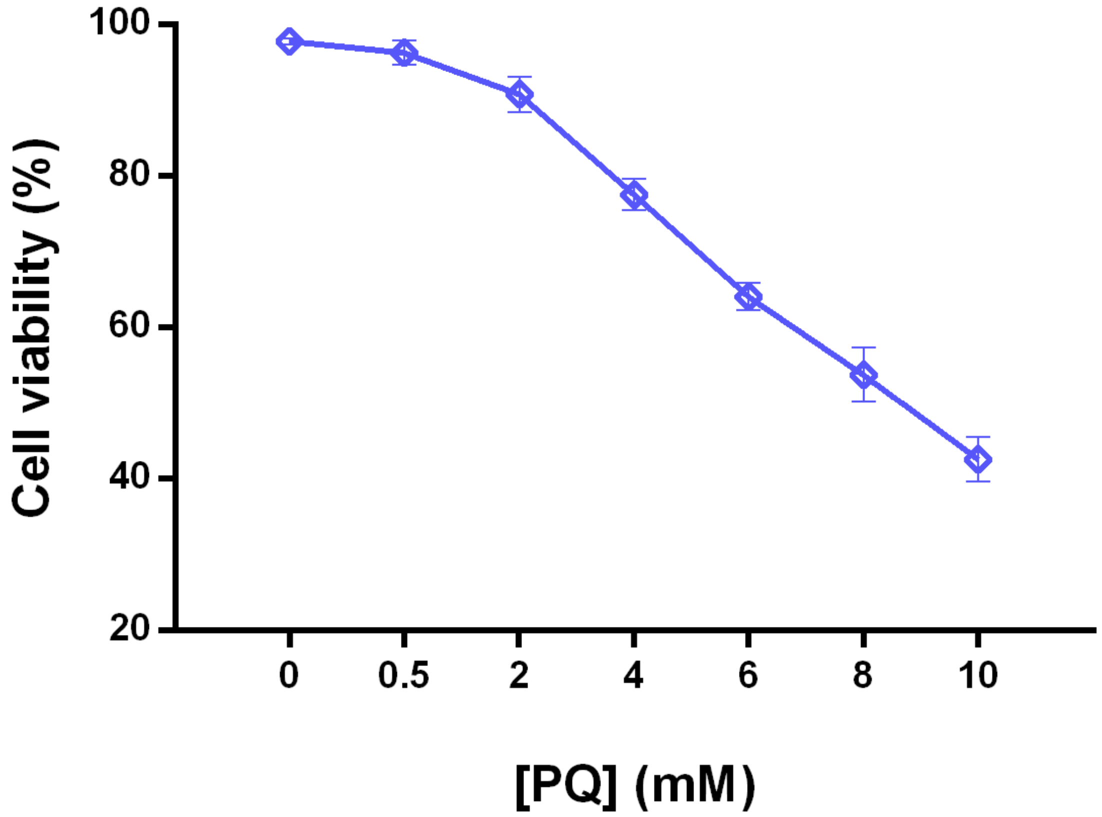

Supplementary Figure 32. Cell viability after treated with PQ for 24 h. Cells were pre-treated with 75 μg/mL S@FNPCN-333 and 75 μg/mL C@FNPCN-333 for 2 h. n=5, mean ± s.d.

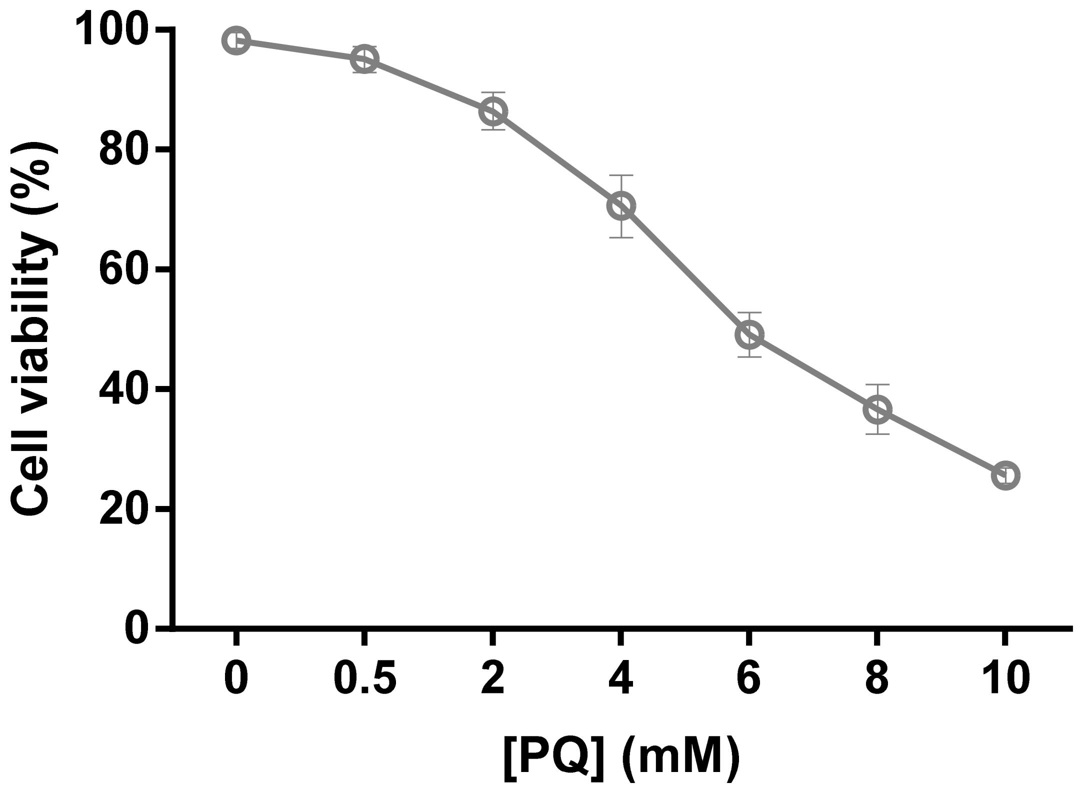

Supplementary Figure 33. Cell viability after PQ treatment. Cells are pre-treated with 75 μg/mL SC@FNPCN-333 for 2 h at 4 °C. n=5, mean ± s.d.

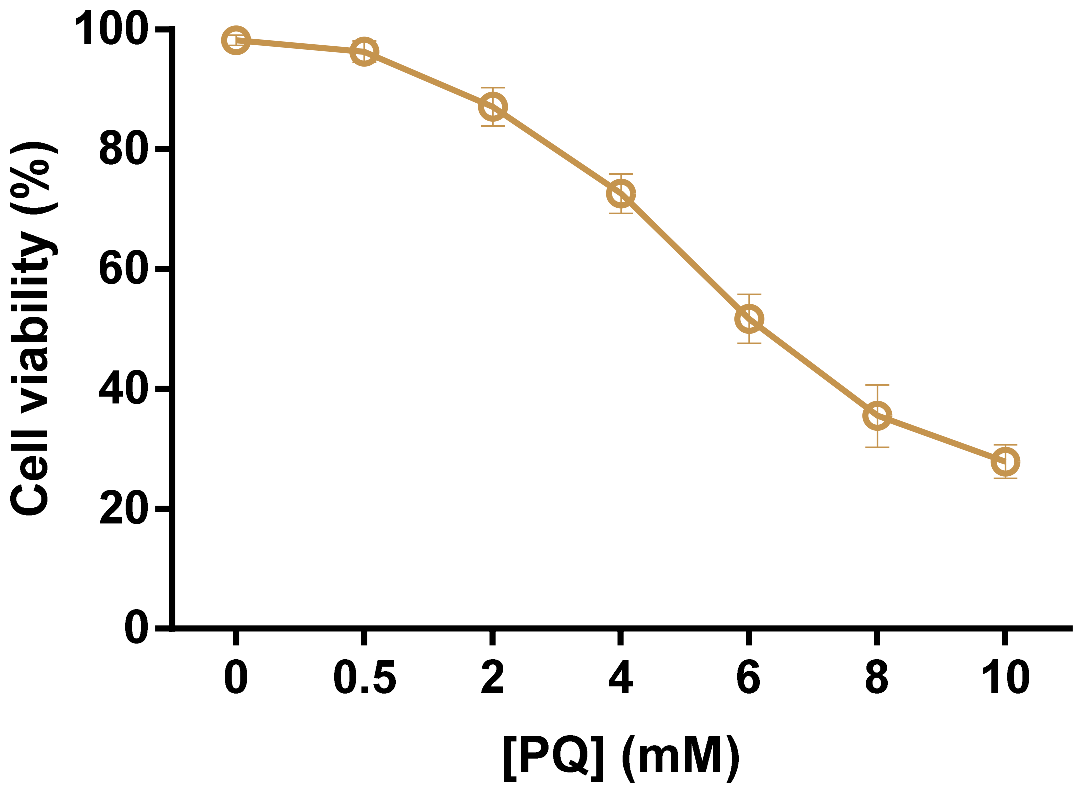

Supplementary Figure 34. Cell viability after PQ treatment. Cells are pre-treated with amiloride for 20 minutes, followed by 75 μg/mL SC@FNPCN-333 and amiloride for 2 h at 37 ^{o}C. n=5, mean ± s.d.

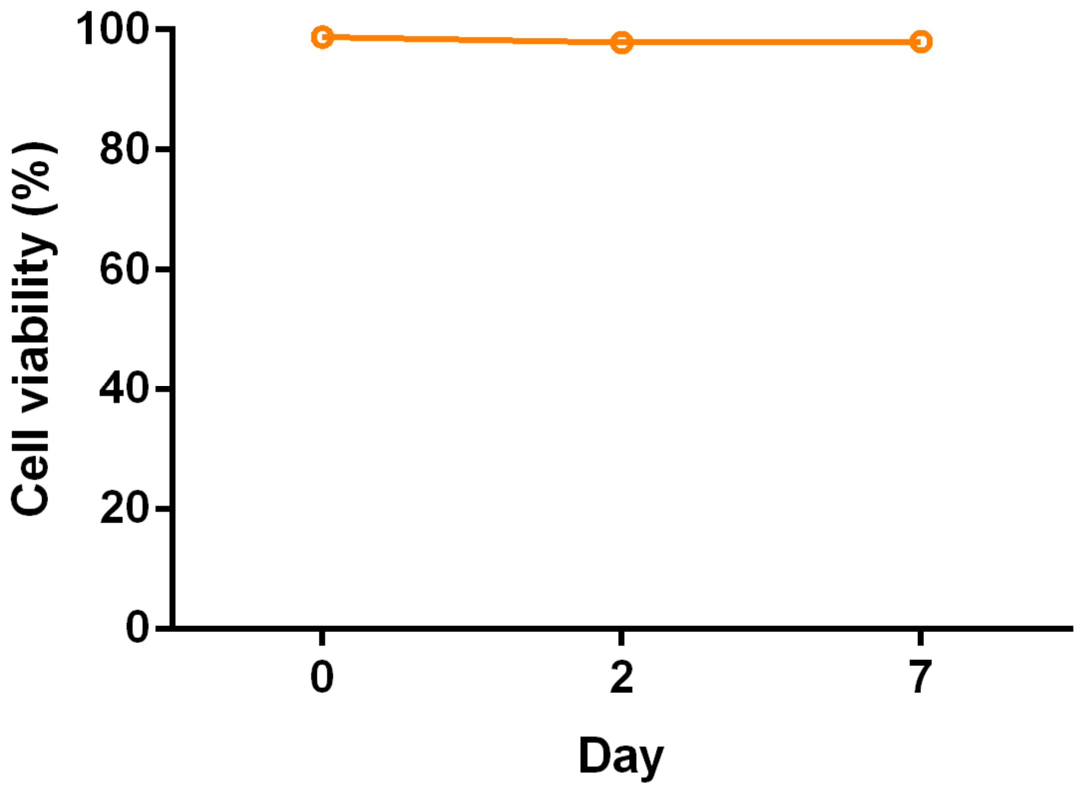

Supplementary Figure 35. Cell viability at day 0, 2 and 7 with SC@FNPCN-333 pretreatment and cultured in PQ free DMEM media at 37°C. n=5, mean ± s.d.

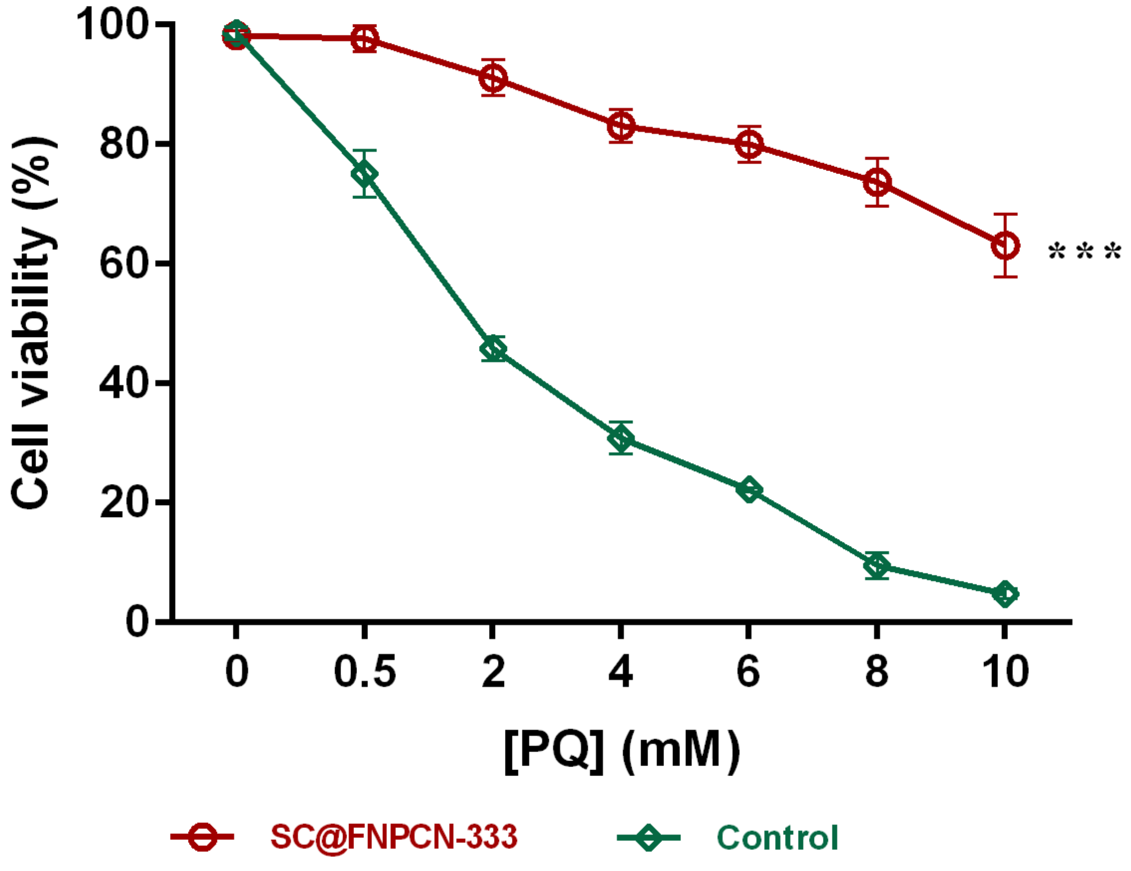

Supplementary Figure 36. NIH3T3 cell viability upon 24 h treatment with different concentrations of PQ with (wine) and without (dark green) 75 µg/mL SC@FNPCN-333 2 h pretreatment. n=5, mean ± s.d. *** represents P≤0.001.

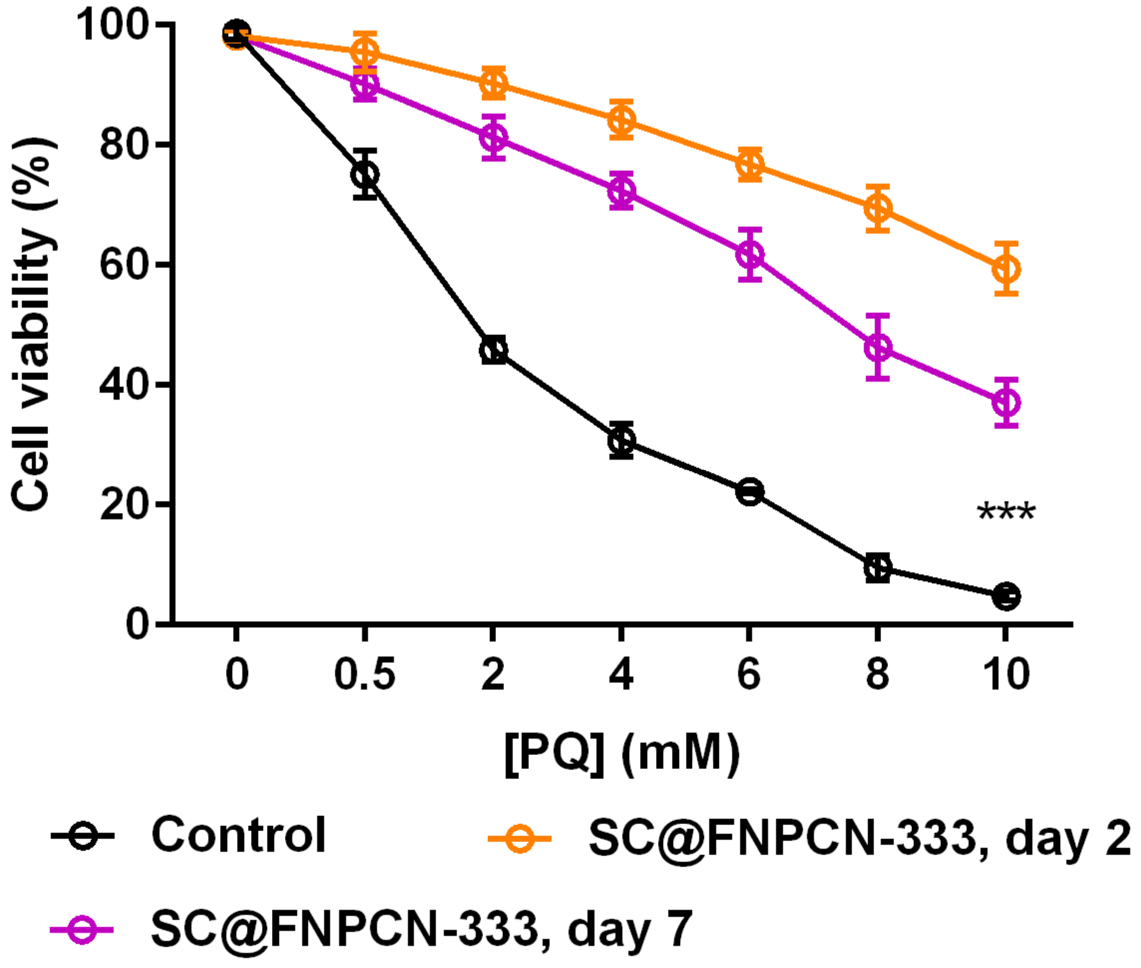

Supplementary Figure 37. Long-term persistence of the protective effect of SC@FNPCN-333 on NIH3T3 cells. Cells were pretreated with SC@FNPCN-333 (75 µg/mL, 2 h), washed and cultured for 2 and 7 days. Cells were then treated with PQ (24 h incubation) and cell viability was quantified. n=5, mean ± s.d. * represents P≤0.001.**

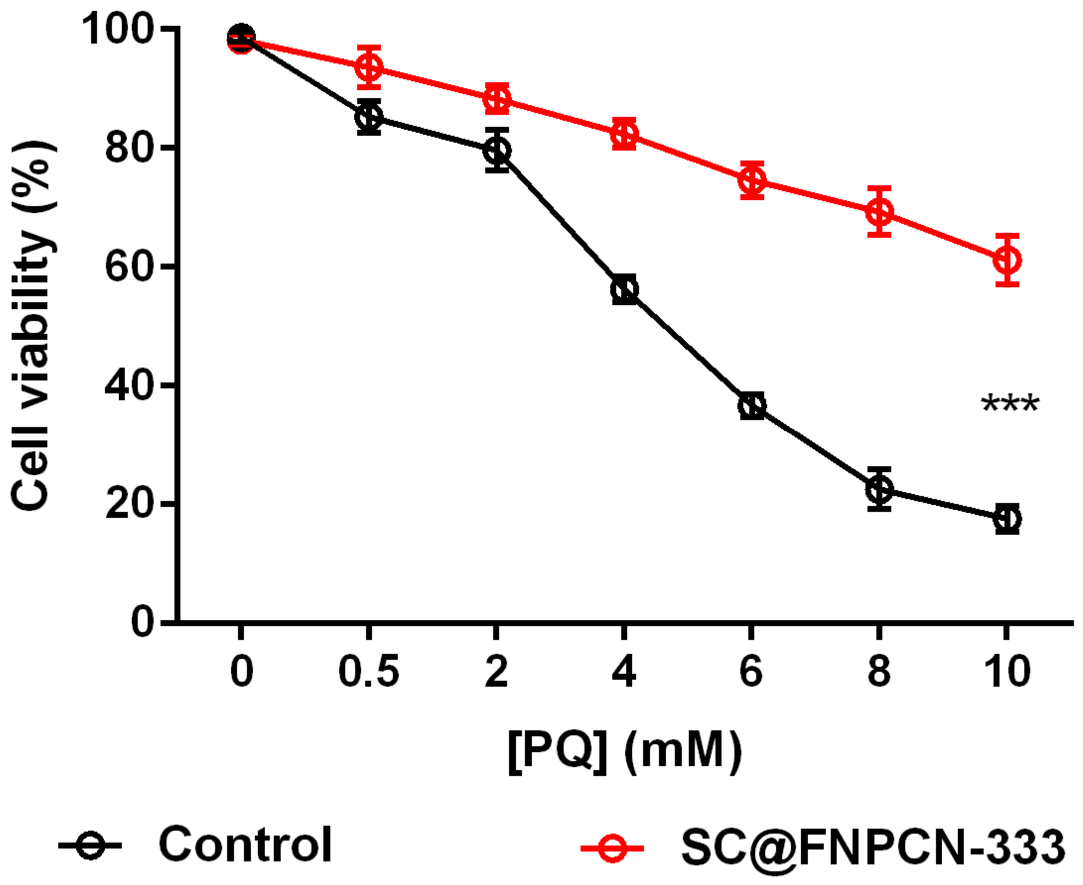

Supplementary Figure 38. **HDF cell viability upon 24 h treatment with different concentrations of PQ with (wine) and without (dark green) 75 μg/mL SC@FNPCN-333 2 h pretreatment. n=5, mean ± s.d. *** represents P≤0.001.**

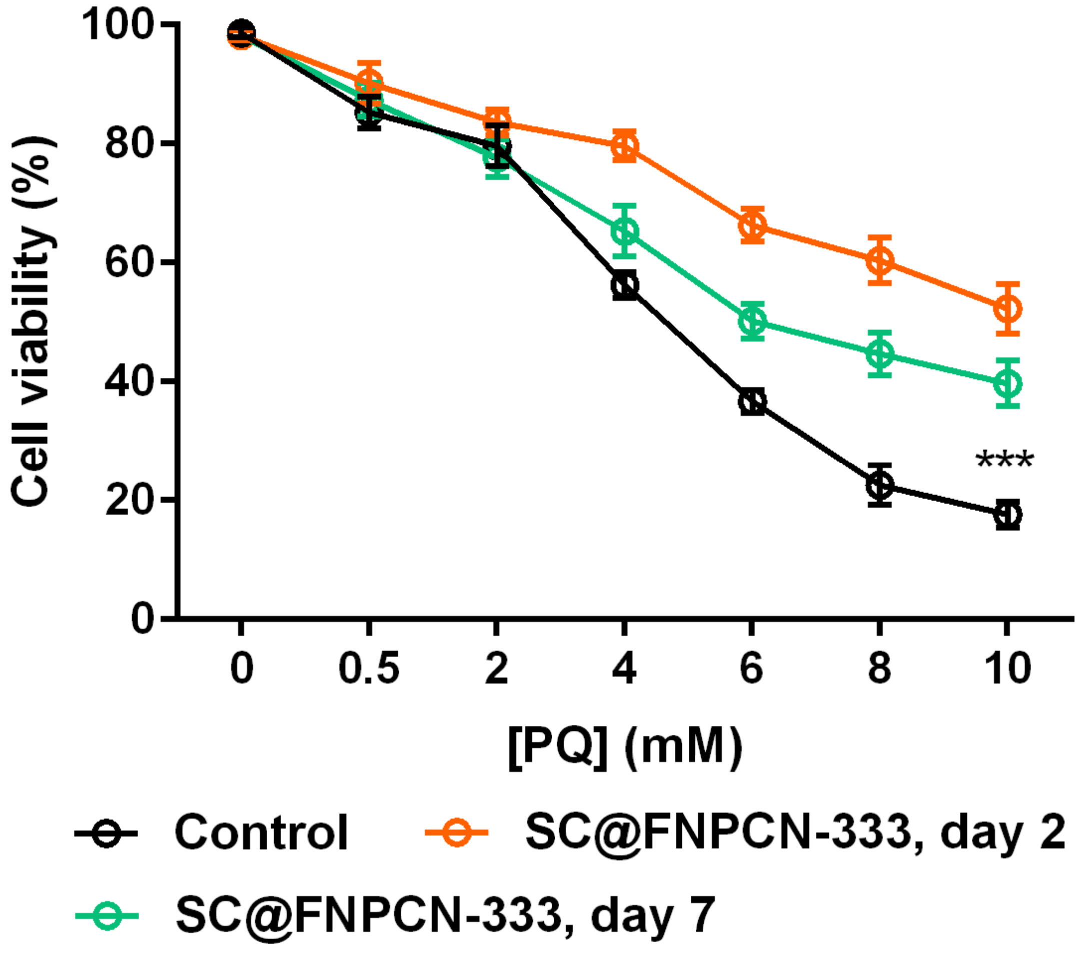

Supplementary Figure 39. Long-term persistence of the protective effect of SC@FNPCN-333 on HDF cells. Cells were pretreated with SC@FNPCN-333 (75 µg/mL, 2 h), washed and cultured for 2 and 7 days. Cells were then treated with PQ (24 h incubation) and cell viability was quantified. n=5, mean ± s.d. * represents P≤0.001.**

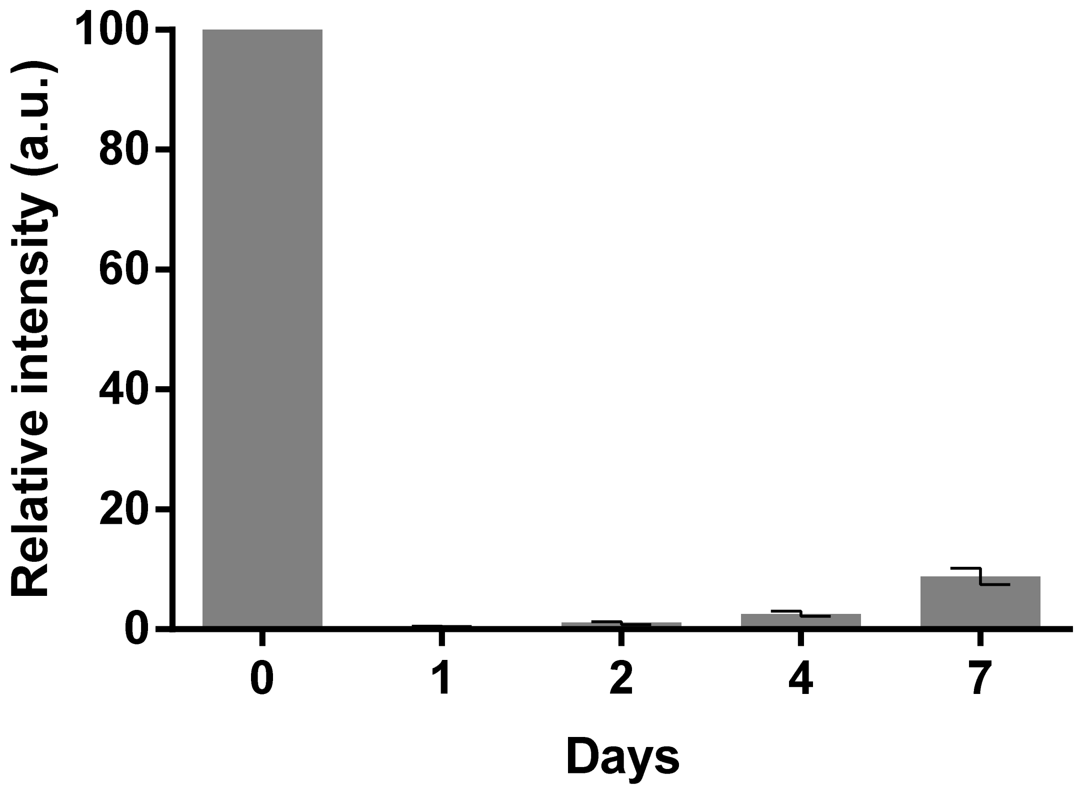

Supplementary Figure 40. Enzyme leaching of SC@FNPCN-333 soaking in pH=5 buffer. 100 μg SC@FNPCN-333 was suspended in 1 mL buffer at 25°C. The leaching amount is determined by BCA assay by measuring the protein concentration of the supernatant on different time point. Day 0 is a measurement of 100 μg/mL protein solution. The leaching amount on day 7 is calculated to be 8.9 μg/mL. n=3, mean ± s.d.

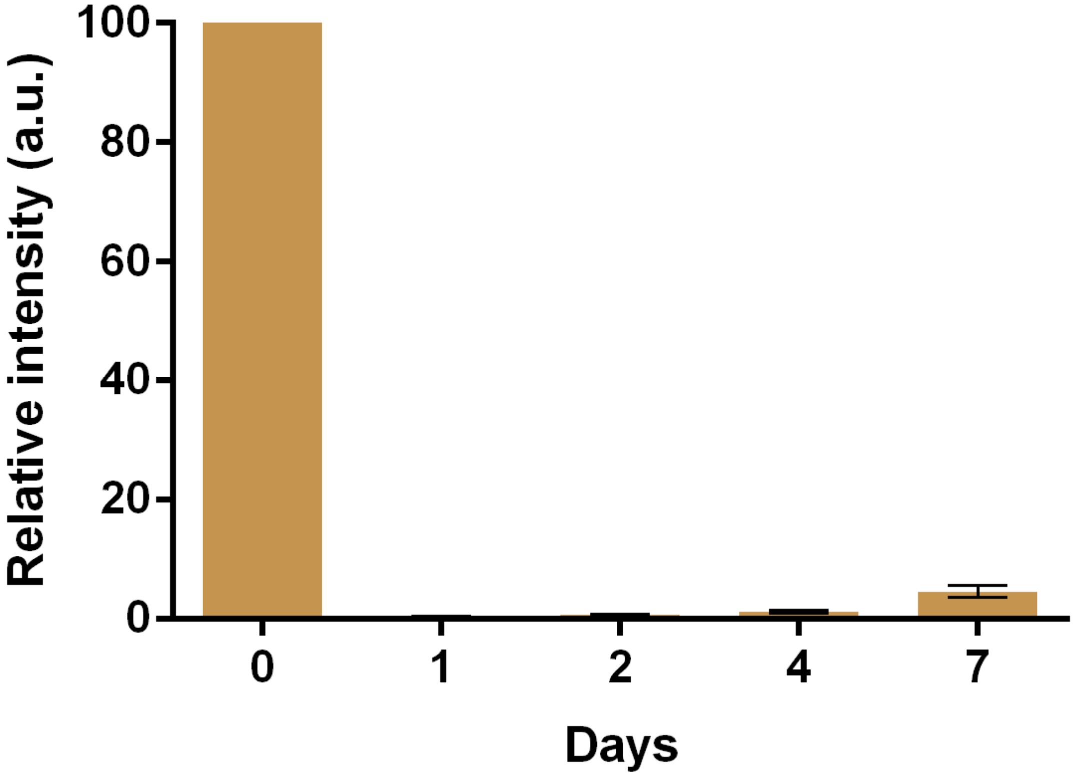

Supplementary Figure 41. Enzyme leaching of SC@FNPCN-333 soaking in pH=7.4 buffer. 100 µg SC@FNPCN-333 was suspended in 1 mL buffer at 25°C. The leaching amount is determined by BCA assay by measuring the protein concentration of the supernatant on different time point. Day 0 is a measurement of 100 µg/mL protein solution. The leaching amount on day 7 is calculated to be 4.6 µg/mL. n=3, mean ± s.d.

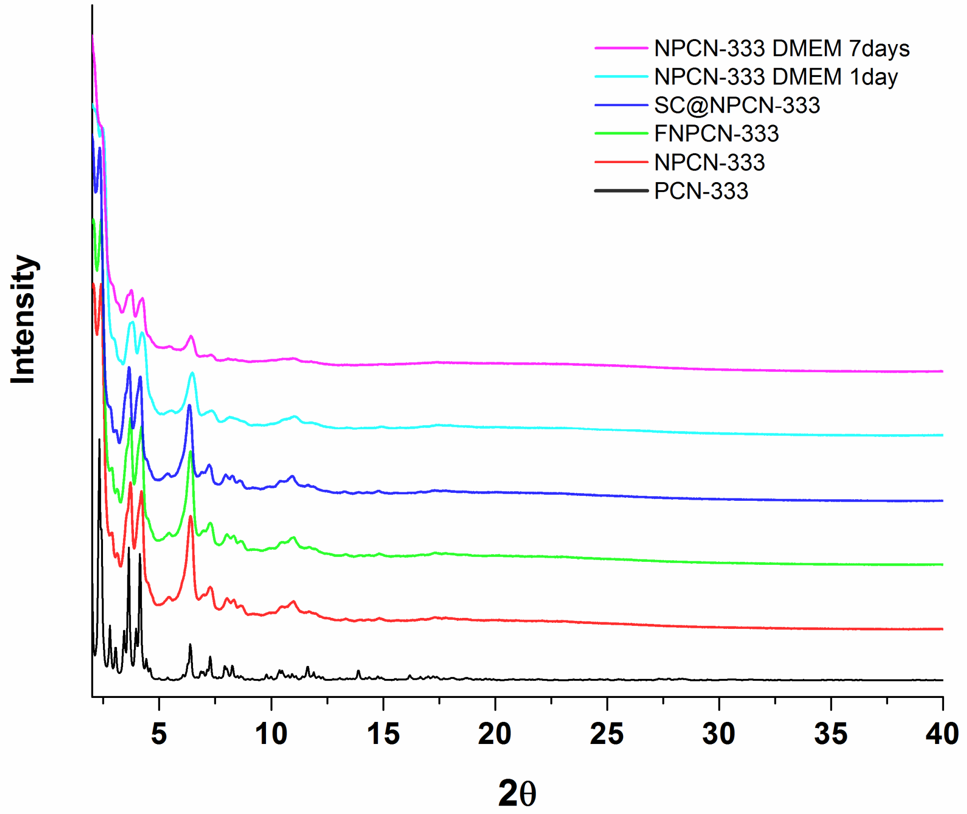

Supplementary Figure 42. Powder X-ray diffraction patterns (2θ from 2 to 40 degree) of microscale PCN-333 (black); NPCN-333 (red); FNPCN-333 (green); SC@FNPCN-333 (blue); NPCN-333 soaked in DMEM for 1 day (cyan); NPCN-333 soaked in DMEM for 7 days (magenta).

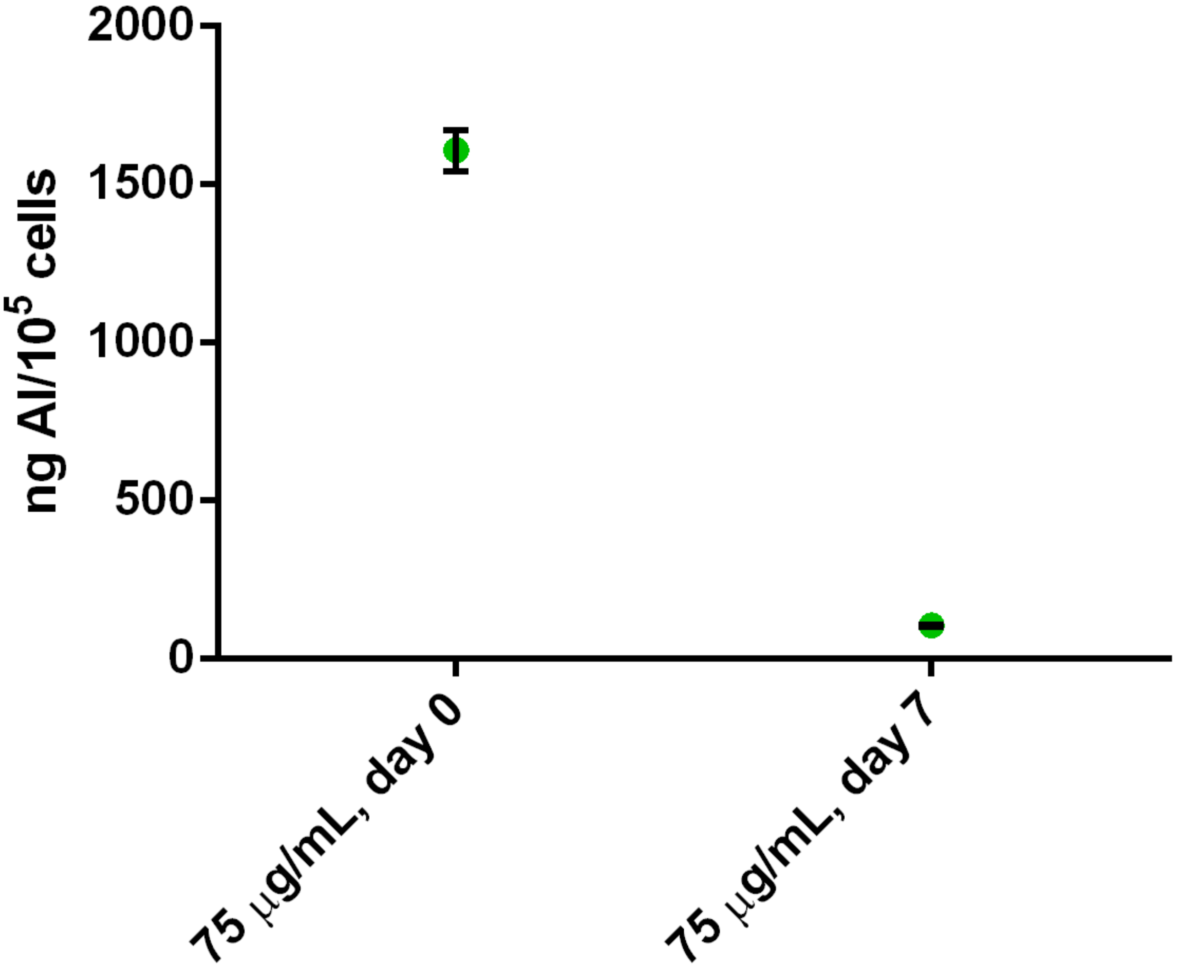

Supplementary Figure 43. Intracellular Al content determined by ICP at day 0 and day 7. Cells were treated with 75 μg/mL SC@FNPCN-333 for 2 h on day 0. n=3, mean ± s.d.

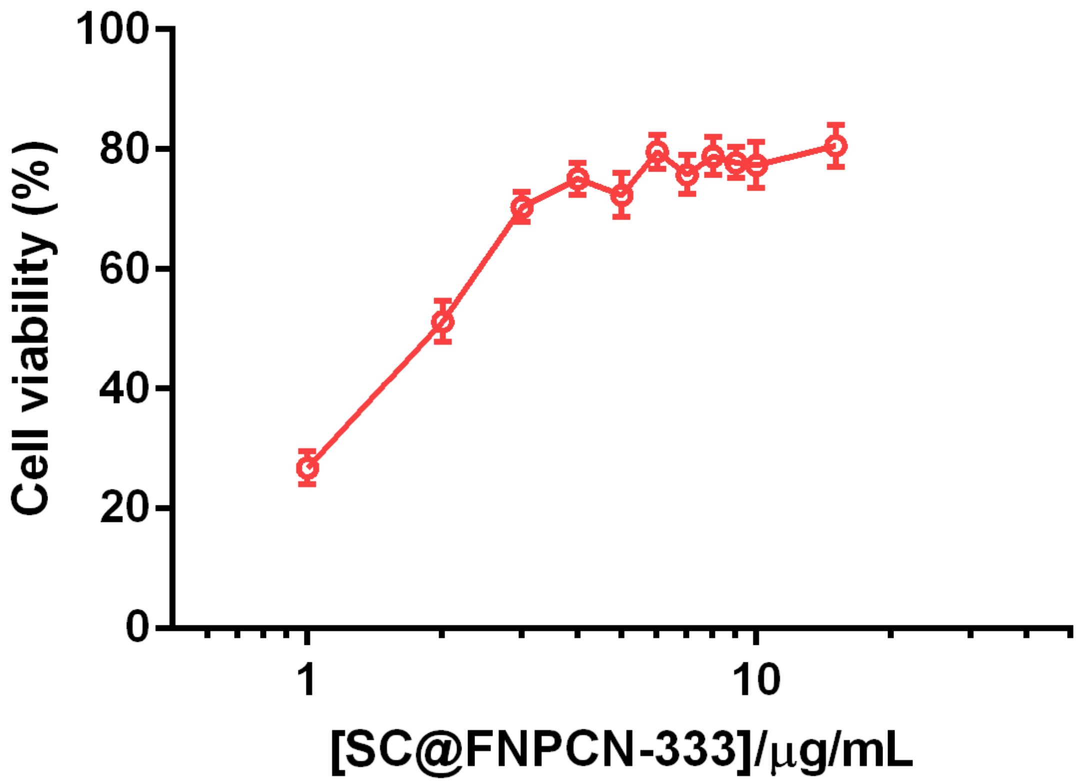

Supplementary Figure 44. Cell viability after 10 mM PQ treatment for 24 h. Cells were pretreated with different concentrations of SC@FNPCN-333 for 2 h. n=5, mean ± s.d.

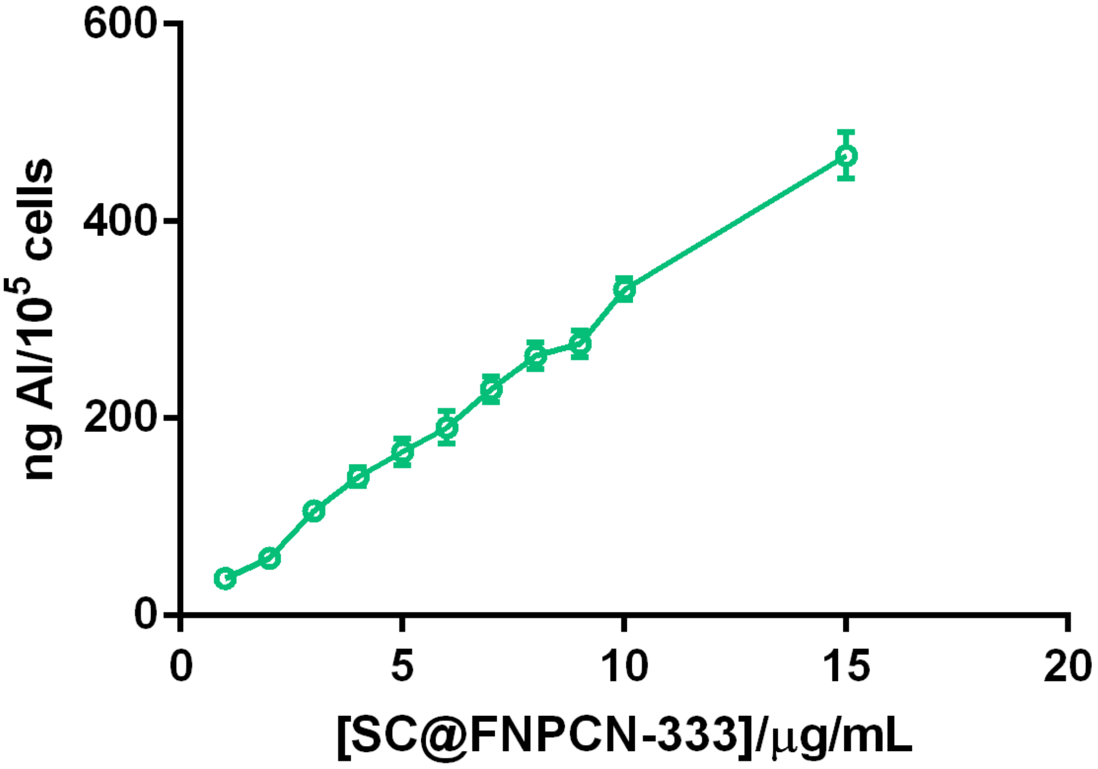

Supplementary Figure 45. ICP analysis of intracellular Al content after treatment with different concentrations of SC@FNPCN-333 for 2 h. n=5, mean ± s.d.

Supplementary References

1 Feng, D. *et al.* Stable metal-organic frameworks containing single-molecule traps for enzyme encapsulation. *Nat. Commun.* **6,** 6979 (2015).

2 Feng, D. *et al.* Kinetically tuned dimensional augmentation as a versatile synthetic route towards robust metal-organic frameworks. *Nat. Commun.* **5**, 5723 (2014).

3 Najjar, K., Erazo-Oliveras, A., Brock, D. J., Wang, T.-Y. & Pellois, J.-P. An L- to D-amino acid conversion in the cell penetrating peptide dfTAT influences proteolytic stability, endocytic uptake, and endosomal escape. *J. Biol. Chem.* **292***,* 847-861 (2016).

4 Schindelin, J. *et al.* Fiji: An open-source platform for biological-image analysis. *Nat. Methods* **9**, 676-682 (2012).

5 Schindelin, J., Rueden, C. T., Hiner, M. C. & Eliceiri, K. W. The ImageJ ecosystem: An open platform for biomedical image analysis. *Mol. Reprod. Dev.* **82**, 518-529 (2015).

6 Byun, M.-S., Jeon, K.-I., Choi, J.-W., Shim, J.-Y. & Jue, D.-M. Dual effect of oxidative stress on NF-[kappa]B activation in HeLa cells. *Exp. Mol. Med.* **34**, 332-339 (2002).

7 Chernyak, B. V. *et al.* Production of reactive oxygen species in mitochondria of HeLa cells under oxidative stress. *Biochim. Biophys. Acta* **1757**, 525-534 (2006).

8 Pallepati, P. & Averill-Bates, D. A. Activation of ER stress and apoptosis by hydrogen peroxide in HeLa cells: Protective role of mild heat preconditioning at 40°C. *Biochim. Biophys. Acta* **1813**, 1987-1999 (2011).
